# Profiling of extracellular vesicles of metastatic urothelial cancer patients to discover protein signatures related to treatment outcome

**DOI:** 10.1002/1878-0261.13288

**Published:** 2022-08-12

**Authors:** Kristina Viktorsson, Petra Hååg, Carl‐Henrik Shah, Bo Franzén, Vasiliki Arapi, Karin Holmsten, Per Sandström, Rolf Lewensohn, Anders Ullén

**Affiliations:** ^1^ Department of Oncology‐Pathology Karolinska Institutet Solna Sweden; ^2^ Department of Pelvic Cancer, Genitourinary Oncology and Urology Unit Karolinska University Hospital Solna Sweden; ^3^ Department of Oncology Capio Sankt Görans Hospital Stockholm Sweden; ^4^ Theme Cancer, Medical Unit Head and Neck, Lung, and Skin Tumours, Thoracic Oncology Center Karolinska University Hospital Solna Sweden

**Keywords:** biomarkers, early treatment response, extracellular vesicles, metastatic urothelial cancer

## Abstract

The prognosis of metastatic urothelial carcinoma (mUC) patients is poor, and early prediction of systemic therapy response would be valuable to improve outcome. In this exploratory study, we investigated protein profiles in sequential plasma‐isolated extracellular vesicles (EVs) from a subset of mUC patients treated within a Phase I trial with vinflunine combined with sorafenib. The isolated EVs were of exosome size and expressed exosome markers CD9, TSG101 and SYND‐1. We found, no association between EVs/ml plasma at baseline and progression‐free survival (PFS). Protein profiling of EVs, using an antibody‐based 92‐plex Proximity Extension Assay on the Oncology II^®^ platform, revealed a heterogeneous protein expression pattern. Qlucore bioinformatic analyses put forward a protein signature comprising of SYND‐1, TNFSF13, FGF‐BP1, TFPI‐2, GZMH, ABL1 and ERBB3 to be putatively associated with PFS. Similarly, a protein signature from EVs that related to best treatment response was found, which included FR‐alpha, TLR 3, TRAIL and FASLG. Several of the markers in the PFS or best treatment response signatures were also identified by a machine learning classification algorithm. In conclusion, protein profiling of EVs isolated from plasma of mUC patients shows a potential to identify protein signatures that may associate with PFS and/or treatment response.

## Graphical abstract

Platinum‐resistant metastatic urothelial cancer (mUC) patients have limited treatment options and short progression‐free survival (PFS), calling for treatment‐guiding biomarkers. We performed multiplex protein profiling of extracellular vesicles (EVs) from plasma of mUC patients treated within Vinsor, a Phase I trial. We analysed EV protein signatures in relation to treatment response by computerised tomography and PFS.

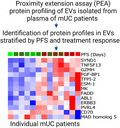

**Abbreviations**
5′‐NT: 5′‐nucleotidase
ABL1: tyrosine‐protein kinase ABL1
ANOVA: analysis of variance
ARID1A: AT‐rich interactive domain‐containing protein 1A
bFGF: basic fibroblast growth factor
BMs: biomarkers
BRAF: proto‐oncogene B‐Raf
BRCA1: breast cancer type 1 susceptibility
CA9: carbonic anhydrase 9
CAIX: carbonic anhydrase IX
CD73: cluster of differentiation 73
CD9: cluster of differentiation 9
cfDNA: cell‐free circulating DNA
CR: complete response
CT: computerised tomography
ctDNA: circulating tumour DNA
DDR: DNA damage response
EDTA: ethylenediaminetetraacetic acid
EGFR: epidermal growth factor receptor
ER: endoplasmic reticulum
ERBB2: erb‐b2 receptor tyrosine kinase 2
ERBB3: erb‐b3 receptor tyrosine kinase 3
ESCRT: endosomal sorting complexes required for transport
EVs: extracellular vesicles
FADD: FAS‐associated protein with death domain
FASLG: FAS ligand
FDA: Food and Drug Administration
FGF2: fibroblast growth factor 2
FGF‐BP1: fibroblast growth factor‐binding protein 1
FGFR: fibroblast growth factor receptor
FR‐alpha: folate receptor alpha
FR‐gamma: folate receptor gamma
GZMH: granzyme H
ICIs: immune checkpoint inhibitors
IFNGR1: IFN‐gamma‐R1
IR: infrared
ISEV: International Society for Extra Cellular Vesicles
LOD: limit of detection
LYN: tyrosine‐protein kinase Lyn
MAD homolog 5: mothers against decapentaplegic homolog 5
MS: mass spectrometry
mUC: metastatic urothelial cancer
MVBs: multivesicular bodies
NF1: neurofibromin
NPX: normalised protein expression
NTA: nanoparticle tracking analysis
PBS: phosphate‐buffered saline
PD: progressive disease
PD‐1: programmed cell death protein 1
PD‐L1: PD‐1 ligand 1
PEA: proximity extension assay
PFS: progression‐free survival
PIK3CA: phosphatidylinositol 4,5‐bisphosphate 3‐kinase catalytic subunit alpha isoform
PR: partial response
*PVRL4*
: poliovirus receptor‐related 4
RAF 1: RAF proto‐oncogene
RB1: retinoblastoma‐associated protein
RECIST 1.1: Response Evaluation Criteria In Solid Tumours version 1.1
RIPA: radioimmunoprecipitation assay
RSPO3: R‐spondin‐3
SD: stable disease
SDS: sodium dodecyl sulfate
SEC: size‐exclusion chromatography
sEVs: small EVs
SYND‐1/SDC: syndecan‐1
TFPI‐2: tissue factor pathway inhibitor 2
TKIs: tyrosine kinase inhibitors
TLR3: toll‐like receptor 3
TNFSF13: tumour necrosis factor ligand superfamily member 13
TP53: tumour protein p53
TRAIL: TNF‐related apoptosis‐inducing ligand
Trop‐2: Trophoblast cell surface antigen 2
TSG101: tumour susceptibility 101
UC: urothelial carcinoma
XGBoost: Extreme Gradient Boosting

## 1 Introduction

For decades, metastatic urothelial cancer (mUC) patients have in first‐line been treated with platinum‐based combination chemotherapy [1, 2]. In platinum‐progressive patients, the microtubule inhibitor vinflunine is approved in the EU, whereas taxanes are often used in the United States [3]. Recently, systemic immunotherapies targeting program cell death protein 1 (PD‐1) or PD‐Ligand 1 (PD‐L1), that is immune checkpoint inhibitors (ICIs), have become part of the standard of care for mUC [4, 5]. Thus, for platinum‐progressive patients, several different PD‐L1‐ or PD‐1‐targeting antibodies are approved, irrespective of tumour PD‐L1 expression level [6]. In patients' ineligible for cisplatin, pembrolizumab and atezolizumab are first‐line alternatives to carboplatin‐based chemotherapy but limited to patients whose tumours have a high PD‐L1 expression level [6]. Beyond ICIs, antibody–drug conjugates targeting Nectin‐4 [also known as poliovirus receptor‐related (PVRL4)] [7, 8] and Trophoblast cell surface antigen 2 (Trop‐2) [9], as well as tyrosine kinase inhibitors (TKIs) targeting fibroblast growth factor receptors (FGFRs) [10, 11], have recently been granted approval by Food and Drug Administration (FDA). Clearly, we are in a time of a rapidly changing and more complex treatment landscape for mUC. How to best select, sequence and combine these precision cancer medicine treatments with different modes of action remains challenging and is an area of increasing importance for patient selection. Accordingly, there is a need for predictive biomarkers (BMs) to optimise treatment sequencing. Genomic or taxonomic subtyping, tumour mutation burden, PD‐L1 expression and alteration of DNA damage response (DDR)‐regulating genes are some examples of BMs, which have been suggested to hold predictive value in advanced urothelial carcinoma (UC) [12, 13, 14].

Patients with progressive platinum‐resistant mUC have poor prognosis and display heterogeneous responses to subsequent treatments calling for early treatment evaluation approaches. Analyses of tumour response markers in liquid biopsies, for example blood, are advantageous for such early treatment evaluation as liquid biopsies are easily accessible and may also capture tumour heterogeneity. Indeed, analyses of circulating tumour DNA (ctDNA)/ cell‐free circulating DNA (cfDNA) are reported in UC and mUC. Thus, mutations in, for example, tumour *protein p53 (TP53)*, *phosphatidylinositol‐4,5‐bisphosphate 3‐kinase catalytic subunit alpha (PIK3CA)*, *erb‐b2 receptor tyrosine kinase 2 (ERBB2)*, *fibroblast growth factor receptor 3 (FGFR3)*, *AT‐rich interaction domain 1A (ARID1A)*, *epidermal growth factor receptor (EGFR)*, *Neurofibromin 1 (NF1)*, *retinoblastoma‐associated protein (RB1)*, *breast cancer type 1 susceptibility (BRCA1)*, *proto‐oncogene B‐Raf (BRAF)* and *RAF proto‐oncogene (RAF1)* have been revealed [15, 16]. However, response to a given therapy is also influenced by tumour RNA and protein signalling alterations, which are not completely captured in ctDNA/cfDNA analyses, thus calling for additional analytical methods on liquid biopsies.

Extracellular vesicles (EVs) cargo miRNA, mRNA and membrane and cytosolic proteins partly resembling their cell of origin and therefore in context of tumours offer a source of BMs [17, 18, 19, 20, 21]. Moreover, EVs may influence signalling within the tumour *per se* and the interplay with the tumour microenvironment [17, 18, 20, 21, 22]. EVs may have different sizes, and the International Society for Extracellular Vesicles (ISEV) classifies them into small EVs (sEVs) (< 100 to ~ 200 nm) and medium/large EVs (> 200 nm) [23]. A particular type of sEVs is those formed via the endosome system, that is exosomes, which are released by viable cells and contain biomolecules from several cellular compartments [18, 21].

EVs from UC cell lines have been demonstrated to influence tumour‐associated signalling, via, for example, metabolic or immune regulation, and EVs isolated from plasma or urine of UC patients constitute a potential source of BMs [19, 24, 25, 26, 27, 28, 29, 30, 31, 32, 33, 34, 35, 36, 37, 38, 39]. Although mass spectrometry (MS) followed by bioinformatics is a common approach for EV proteome analyses, such methodology may not always be feasible as the amount of EVs that can be isolated from plasma varies due to dynamic processes. Here, the multiplex antibody‐based platform, proximity extension assay (PEA) with high sensitivity offer a way forward. Indeed, the PEA technology has been applied for analysing protein cargo of EVs from different body fluids [40] and to reveal prognostic BMs of glioma and glioblastoma cancer patients in plasma, for example syndecan‐1 (SYND‐1 also known as SDC1) [41].

Previously, in our Phase I trial Vinsor, we investigated the combination of vinflunine and the pan‐TKI sorafenib in second‐line patients with platinum‐progressive mUC [42]. In this exploratory study, we applied PEA analytics on EVs isolated from plasma of a subset of the patients from the Vinsor trial treated at the Karolinska University Hospital (*n* = 13). The aim was to explore whether early alterations in EV protein expression could be associated with subsequent clinical outcome.

## 2 Materials and methods

### 2.1 Patient material and assessment of treatment response

We have earlier conducted and reported a Phase I clinical trial, Vinsor (Eudra‐CT 2011‐004289‐14, NCT01844947), in which patients with platinum‐resistant mUC (*n* = 22) were treated with a combination of vinflunine and sorafenib [42]. In a subset of the patients (*n* = 13), included at the Karolinska University Hospital, Solna, Sweden, sequential plasma samples were collected and used in the present exploratory BM study (Table 1). The Vinsor study was approved by the Swedish Medical Products Agency (151:2012/12127) and the Regional Ethical Review Board in Stockholm (2011/1398‐31/1). All patients signed a written informed consent, and the biobanking of plasma from the patients obtained approval from Stockholms Medicinska Biobank (BbK‐728).

In the Vinsor study, computerised tomography (CT) scans were carried out prior to starting the first, second and third treatment cycle and thereafter prior to every other treatment cycle. CT scans were evaluated with Response Evaluation Criteria In Solid Tumours version 1.1 (RECIST 1.1). PFS was defined as time from study inclusion to either progression according to RECIST 1.1 or death. Two patients (pat. #110 and pat. #112) did not complete the first cycle of the study treatment and hence were per protocol excluded from response evaluation per RECIST 1.1 (Table 1). The treatment response in this exploratory study was analysed as early response, that is after one treatment cycle (CT1), and best response, that is the maximum treatment response seen at any time point during the treatment course. The RECIST 1.1 evaluations of the patients are described in Table 1. The methods applied in this study conformed with the standards set by the Declaration of Helsinki. Treatment response was in this study evaluated in a more exploratory way by measuring the sum of the target lesions as a continuous variable compared with baseline measurement (percentage, %). This value was used alongside PFS as an exploratory efficacy endpoint in relation to the protein signature analysis of the EVs.

### 2.2 Plasma sample processing and isolation of EVs

ethylenediaminetetraacetic acid (EDTA) plasma from the mUC patients (Table 1) was collected at baseline, at day 8 and day 21 after treatment initiation, and was stored at −80 °C. EVs were isolated from ~ 3 mL plasma per sample. The plasma was thawed on ice, cleared from cell debris by centrifugation at 720 ***g*** for 5 min (centrifuge Rotina 38, Hettich Lab Instrument AB, Stockholm, Sweden), and subsequently filtered through a 0.22‐μm syringe filter (Acrodisc^®^, Pall Corporations, VWR, Stockholm, Sweden) to take out plasma protein complexes. Due to differences in viscosity of the plasma samples, the number of filters needed for this step differed among the samples. This may have caused trapping of some sample parts in the filter, which potentially may have impacted on the total sample volume and/or number of EVs. Filtered plasma was concentrated on Amicon^®^ ultra‐4 (Ultracel^®^‐3K) centrifugal filters (Merck Chemicals and Life Science AB, Solna, Sweden). About 350–600 μL of the concentrated plasma sample was loaded onto Izon's qEVoriginal 70‐nm size‐exclusion chromatography (SEC) column (Izon Science, Oxford, UK). Captured EVs were eluted from the column using sterile‐filtered (0.22‐μm syringe filter, Acrodisc) phosphate‐buffered saline (PBS) (HyClone™, GE Health Care, Uppsala, Sweden) in 500 μL fractions. In preparatory experiments, exosome‐sized EVs were found in fractions 6–10. In this study, fractions 6–10 were therefore pooled and concentrated by Amicon^®^ ultra‐4 (Ultracel^®^‐3K) centrifugal filters (Merck Chemicals and Life Science AB) and applied for EV protein profiling for most of the samples (see Section 2.3).

### 2.3 Nanoparticle tracking analysis of EV size and estimation of EV amount

To assess particle size and amount in the isolated and pooled fractions 6–10, nanoparticle tracking analysis (NTA) was performed. The samples were assessed by NTA as non‐concentrated pooled EVs (directly after the SEC column isolation) for determination of the size of the EVs as well as of the plasma concentration of EVs. The EV samples were also analysed by NTA after concentration to be able to relate amount of EVs to PEA and western blot analyses. For the NTA assessment of the non‐concentrated samples, fractions 7–10 were pooled and analysed for pat. #110 at day 8. For pat. #107 and pat. #109 at baseline, fraction 8 was measured. For all other samples pooled, fractions 6–10 were analysed. NTA was carried out on the NS300 instrument (NanoSight, Malvern Panalytical, Malvern, UK), and the EV samples were diluted to get around 50–100 particles per frame. The following settings were applied: analysis time 3–5 × 90 s, syringe load 100, camera level 8–16 and threshold for analysis 5–11 depending on the sample character. For baseline samples, the obtained EVs/ml was related to the extracted plasma volume to obtain EVs/ml plasma and plotted against PFS of the patients. The correlation between EVs/ml and the PFS of the patients (Table 1) was analysed with the Pearson coefficient tool integrated in the graphpad prism software (GraphPad Software, Inc., LA Jolla, San Diego, CA, USA). The number of EVs at days 8 and 21 was examined in individual samples and presented as fold increase relative to baseline and plotted against PFS. NTA was also carried out on the pooled and concentrated fractions 6–10 in all patient samples except for the pat. #110, day 8 sample where fractions 7–10 were analysed. The concentrated samples were applied in the PEA and western blot analyses, respectively. The NTA settings were as follows: analysis time 3 × 60 s, syringe load 100, camera level 12–16 and threshold for analysis 5.

### 2.4 Proximity extension assay protein profiling of EV samples

Protein profiling of EV samples was carried out by proximity extension assay (PEA) on the Oncology II^®^ panel (Olink Proteomics AB, Uppsala, Sweden). The Oncology II panel comprises antibody pairs towards 92 proteins, which control not only tumour cell proliferation, migration and cell death signalling but also factors associated with the tumour microenvironment, for example extracellular matrix components, angiogenesis or immune cell signalling (for full list, see https://www.olink.com/content/uploads/2021/09/olink‐oncology‐ii‐validation‐data‐v2.0.pdf and Section 3.2). The PEA profiling was performed by the Clinical Biomarker Facility, Science for Life Laboratory, Uppsala University, Uppsala. The concentrated EV samples (see Section 2.3) were dissolved in 5× Radioimmunoprecipitation assay (RIPA) buffer to a final concentration of 1× RIPA (25 mm Tris/HCl pH 7.4, 150 mm NaCl, 1% NP‐40, 2 mm EDTA, 0.1% sodium dodecyl sulfate (SDS) (all chemicals were obtained from Sigma‐Aldrich, Sweden AB, Stockholm, Sweden)) supplemented with fresh PhosSTOP and cOmplete Mini‐EDTA free addition (both from Roche Diagnostics via Merck). From these EV lysates, 1 μL was applied for the PEA assay. The number of EVs profiled by PEA from the different patient plasma samples ranged from ~ 5 × 10^5^ to 3 × 10^8^. The difference in the number of EVs analysed by PEA profiling was taken into consideration when evaluating the protein expression profiles (see Section 2.5).

For data processing, the Olink Wizard for genex software (MultiD Analyses AB, Gothenburg, Sweden) was used alongside manual curation. The normalised protein expression (NPX) values generated in the assay were applied in the subsequent bioinformatic analyses. For each PEA protein reaction, the vendor has established a lower limit of detection (LOD), described by negative control and three standard deviations obtained when setting up the panel (https://www.olink.com/question/how‐is‐the‐limit‐of‐detection‐lod‐estimated‐and‐handled/). As this is a rather stringent cut‐off, this exploratory study of EVs also included values for samples that were below LOD into the Qlucore bioinformatics analyses. Thus, proteins limited to those that had an expression above RIPA control (reaction without EV lysate) for the individual markers in > 50% of the baseline samples were included. This generated a data set of 86 markers out of the 92 protein reactions within the Oncology II^®^ panel (Table S1).

### 2.5 Qlucore bioinformatics analyses of PEA protein profiling data

The PEA NPX data were bioinformatically processed using Qlucore Omics Explorer 3.5 (Qlucore AB, Lund, Sweden, https://www.qlucore.com/). The analyses were carried out by first sorting the proteins based on variance. To filter out major differences, subgroups of samples were compared by ANOVA (*P*‐value set to ≤ 0.05 if not otherwise indicated). To reveal outliers and tentative protein signatures in the PEA profiling data, non‐supervised hierarchical clustering and principal component analysis were used, respectively. Hierarchical clustering was also applied to visualise sample similarity with respect to protein expression profiles in EVs in relation to patients' PFS or best treatment response (see Section 2.1). Here, the number of EVs, that is ‘amount of EV’ analysed by PEA from each plasma sample, was used for normalisation with EV values calculated from the NTAs, which is regarded as a reliable method for assessing amount of EVs [43]. The integrated tool, ‘Elimination factor(s)’, in the Qlucore Omics Explorer software was used, which corrects the part of the data that can be explained by a given factor (in this study, number of EVs analysed by PEA). The integrated tool sets up a general linear model (GLM, based on *F*‐test), which postulate that data can be modelled by the ‘Elimination factors’ only (in this work by number of EVs analysed by PEA; null hypothesis) or that ‘Elimination factors’ and the test factor (in this work either PFS or best treatment response; alternative hypothesis) together allow data modelling. Therefore, this method indorses that the EV protein profiles will be associated with PFS or best treatment response and not be a result of different amounts of EVs being studied in the analysed samples. The PEA data were also explored using a machine learning algorithm XGBoost (Extreme Gradient Boosting, Qlucore integrated tool; see https://xgboost.readthedocs.io/en/latest/#) to build classifier models. These models were constructed based on all proteins (*n* = 86; see Section 2.4) and made to compare patients with short (≤ 138 days) or long (> 138 days) PFS or best treatment response [partial response (PR) vs stable disease (SD) or progressive disease (PD)], respectively.

The individual protein expression levels revealed in the EV samples were further analysed and visualised using the graphpad prism software (GraphPad Software, Inc.). For analyses related to the PFS‐associated protein signature in EVs, the patients were grouped into short (≤ 138 days) or long (> 138 days) PFS as an arbitrary cut‐off giving equal group sizes and analysed for their protein NPX values (see Section 3.4). This was also done after normalising the linearised NPX values for the number of EVs analysed by the PEA. Data are presented as log 2‐transformed values. Please note that the sample from pat. #114 was for reasons related to low amount of EVs excluded in some of the analyses presented in Sections 3.4 and 3.5. For linear regression analyses, the NPX values of the different PEA reactions were linearised and analysed in relation to PFS or best treatment response without normalising for number of EVs used in the PEA analyses (see Sections 3.4 and 3.5). The inbuilt statistical functions for *t*‐test and for linear regression via the Pearson correlation coefficient tool in the graphpad Software were applied with the obtained *P*‐values indicated.

### 2.6 Western blot analyses of EVs

EV samples from pooled and concentrated fractions were dissolved in RIPA buffer (see Section 2.4) and used for the western blot profiling. In some of the western blot analyses, a total cell extract from the UC cell line J82 (ATCC, distributed by LGC Standards, Teddington, UK) or the non‐small‐cell lung cancer cell line PC9 (Sigma‐Aldrich) was used as positive controls. Prior to western blot analyses, sample buffer and reducing agent (Invitrogen NuPAGE^®^, Thermo Fisher Scientific, Stockholm, Sweden) were added to the samples. The number of EVs (as determined by NTA) loaded in each line of the blots is presented in the figures. Samples were resolved on Bis‐Tris gels (4–12%) with MES buffer (all reagents from Invitrogen). Proteins were blotted to nitrocellulose membranes (LI‐COR GmbH, Bad Homburg, Germany) using 10% methanol in the transfer buffer and with the membranes subsequently blocked in TBST: Intercept^®^ Blocking buffer 1 : 1 (LI‐COR). Membranes were probed with primary antibodies: anti‐CD9 (#13403) and anti‐calnexin (#2433) (both from Cell Signalling Technology, BioNordika AB, Stockholm, Sweden, dilution 1 : 1000); recombinant anti‐tumour susceptibility 101 (TSG101) antibody [EPR71(B)] (#ab125011; Abcam, Cambridge, UK, dilution 1 : 500); and anti‐syndecan‐1/CD138 antibody (#36–2900, dilution 1:500) and anti‐Granzyme H (#PA5‐83074, dilution 1:200) (both from Thermo Fisher Scientific). Primary antibody binding was visualised by 1:15000 dilutions of 800CW IRDYe^®^ Goat anti‐Rabbit (#926–32211) or IRDye^®^ 680RD Donkey‐anti Mouse (#926–68072) in TBST (both antibodies from LI‐COR). The resulting antibody bindings were monitored on the Odyssey^®^ Sa Infrared Imaging System (LI‐COR).

## 3 Results

### 3.1 Study outline and patient characteristics

In a subset of the patients included in the Phase I Vinsor trial (*n* = 13), in which a combination of vinflunine and sorafenib was evaluated [42], plasma was collected at baseline, days 8 and 21. In this exploratory study, our aim was to reveal whether protein profiles in EVs isolated from these plasma samples taken during the initial treatment course could predict PFS or were associated with best treatment response. The study outline is given in Fig. S1A. For that purpose, isolated EVs were profiled for protein expression using PEA on the Oncology II^®^ panel (for details, see Section 2.4 and Table S1).

The patient clinical efficacy data are given in Table 1. The analysed patient cohort (median age: 62 years, male:female 8:5) had their primary tumour in the bladder (*n* = 9), renal pelvis (*n* = 2) and ureter (*n* = 2), respectively. Seven patients had undergone surgery of the primary tumour [42]. The site of the metastatic lesions in the patients is given in Table S2 with 9 of the 13 patients having visceral metastasis at the start of treatment. The median number of second‐line treatment cycles was 6 (range 1–16) resulting in a median PFS and overall survival (OS) of 4.6 and 8.4 months, respectively (Table 1). Six patients showed a partial response to the study combination vinflunine plus sorafenib, and a further four patients achieved stable disease according to RECIST 1.1 (Table 1). The decrease in size of the target lesions measured by CT after one treatment cycle (CT1) relative to baseline revealed a heterogeneous response among the patients with all but two patients showing decrease in tumour size (Fig. S1B, top panel). In some patients, response was delayed beyond CT1. Therefore, when assessing the maximal tumour response observed at any treatment cycle, indicated as best CT response, a further tumour size reduction was evident in some but not all of the responding patients (Fig. S1B, top panel). Moreover, analyses of CT1 and best CT response in relation to PFS, respectively, showed a significant correlation to best CT response (*P* = 0.03) but not in relation to CT1 (Fig. S1B, bottom panel).

**Table 1:** Treatment cycles, survival outcome and response data of the metastatic urothelial cancer patients included in the Vinsor trial at the Karolinska University Hospital.

Patient no.	No. of treatment cycles	Progression‐free survival (days)	Overall survival (days)	Sum of target lesions on CT at baseline (mm)^a^	Sum of target lesions on CT after one tx cycle (mm)^a^	Best response (minimal sum of target lesions) on any CT (mm)^b^	Best outcome by RECIST 1.1^c^
101	6	125	188	70	84	72	SD
102	7	200	219	56	50	34	PR
103	6	130	190	46	27	21	PR
105	6	132	379	48	ND^d^	43	SD
106	6	138	179	142	120	85	PR
107	16	483	1252	20	15	4	PR
108	6	119	461	61	65	65	SD
109	3	53	53	44	43	43	SD
110	1	257	257	73	54	54	ND^e^
111	8	155	406	60	51	36	PR
112	1	339	339	50	40	40	ND^e^
113	2	37	82	22	20	20	PD
114	7	151	357	23	20	12	PR

Abbreviations: CR, complete response; CT, computerised tomography; ND, not determined; PD, progressive disease; PR, partial response; RECIST, Response Evaluation Criteria In Solid Tumours; SD, stable disease.

a Value stated is the sum of target tumour lesions on CT in mm. Baseline CT (CT0) was performed 1–28 days prior to start of study treatment. CT1 was completed 1–8 days prior to commencing cycle 2.

b Best CT reflects the minimal sum of target lesions in mm observed at any occasion (within this study) following baseline CT in each patient.

c RECIST v. 1.1 was carried out as in standard procedures with the following outcomes: CR, PR, SD, PD or ND.

d For pat. #105, data from CT after one treatment cycle is missing because no CT was performed during the designated time interval prior to initiating the second treatment cycle (cycle 2).

e Per study protocol Vinsor, the patient did not full fill criteria for response evaluation by RECIST v.1.1.

### 3.2 A heterogeneous amount of small‐sized EVs is found in Vinsor patient plasma samples at baseline and after treatment

**Fig. 1:**
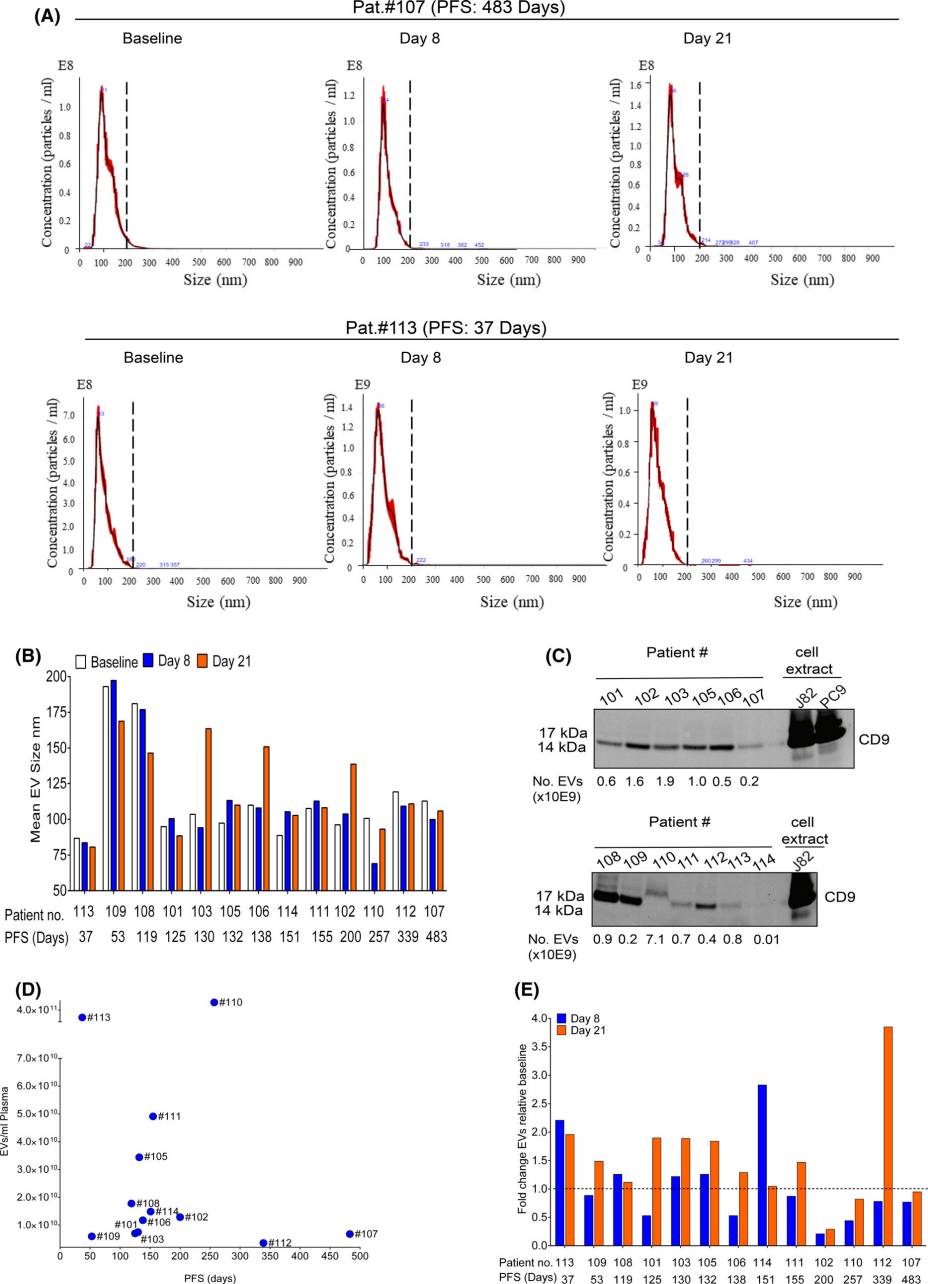
Extracellular vesicles isolated from plasma of metastatic urothelial cancer patients show heterogeneity in size and concentration at baseline and during treatment. Extracellular vesicles (EVs) were isolated from plasma samples at baseline, day 8 and 21. The samples were analysed for particle size (nm) and concentration (EVs/ml) by nanoparticle tracking analysis (NTA) (A, B, D, E) and profiled by western blot (C). (A) NTA histograms of EVs isolated from plasma of metastatic urothelial cancer (mUC) pat. #107 and #113 who had the longest and the shortest progression‐free survival (PFS)  in days, respectively. The dotted vertical line in the graphs is set at 200 nm. NTA histograms for all patients are shown in Fig. S2. Presented data are the mean of three replicate runs on the NTA but from one biological isolate. (B) Quantification of mean particle size (nm). Please note that for pat. #110, pooled fractions 7–10 were analysed at day 8, and for samples from pat. #107 and pat. #109, only fraction 8 only was measured at baseline. The PFS (in days) of the individual patients is stated. The mean size of EVs were obtained from three replicate runs on the NTA of one biological isolate. (C). EVs were profiled for CD9 expression by western blotting. Cell extracts from the urothelial carcinoma cell line J82 and the Non‐small lung cancer cell line PC9 were used as positive controls. The amount of EVs loaded in each sample is stated below the figure. Please note that no normalisation for amount of EVs was performed in these analyses; the presented result just verifies that CD9 is expressed in all the samples but not the relative expression between the samples. Data presented are from one biological isolation of EVs from the plasma samples. (D). The concentration of EVs in individual mUC patient plasma samples at baseline was quantified by NTA, and presented data, EVs/ml, were obtained after adjusting for differences in starting volumes used for isolation of EVs. (E). Fold change in EVs from mUC patient plasma samples at day 8 or 21 relative to baseline is shown. Please note that for (D) and (E), different fractions from the size‐exclusion chromatography (SEC) column were used for generating the data from some patients (see Section 2.3).

The size and the amount of EVs in the different patient samples were characterised using nanoparticle tracking analyses (NTA) (Fig. 1A, Fig. S2). In most of the samples, the EVs had a size smaller than 200 nm as exemplified by pat. #107 (PFS: 483 days) and pat. #113 (PFS: 37 days) (Fig. 1A, Fig. S2). The mean size of the EVs in the entire sample cohort was ~115 nm but with some patients, i.e. pat. #108 and pat. #109, also showing larger sized EVs at baseline (Fig. 1B, Fig. S2). The heterogeneity in EV size was also evident at day 8 and 21 post‐treatment with pat. #102, pat. #103 and pat. #106 showing a switch towards larger‐sized EVs at day 21 (Fig. 1B). For non‐concentrated pat. #107 and pat. #109 baseline samples, only fraction 8 was analysed on NTA (see Section 2.3). This could have influenced the mean size of the EVs; however, the size of the studied vesicles was rather like those of day 8 or 21 where samples from pooled fractions 6–10 were analysed. We also observed smaller sized vesicles at day 8 in pat. #110. Although this can be linked to the molecular character of the sample, for example the cell origin of the EVs and/or treatment response of the patient, it may also be because fractions 7–10 were used in the analyses. In summary, while we observed heterogeneity in the mean size of the EVs in the different patient samples, we did not see any consistent change in the pattern of EV mean size upon treatment. Thus, the isolated EVs from the plasma samples of mUC patients could be considered to be sEVs according to the ISEV definition [23].

The tetraspanin CD9, a category 1 EV marker [23], was expressed in the EV samples both at baseline (Fig. 1C) and at post‐treatment (data not shown) indicating endosomal origin of the EVs. Similarly, the endosomal sorting complexes required for transport (ESCRT) I component TSG101, an EV category 2 marker [23], was present in the EV samples (Fig. S3). Analyses of the endoplasmic reticulum protein calnexin in the baseline samples did not reveal expression suggesting no major cellular protein contamination (Fig. S3). As we did not load equal amount of EVs when analysing these markers due to shortage in sample availability, we detected less expression of CD9 in some samples, for example pat. #107, pat. #111, pat. #113 and pat. #114 (Fig. 1C). Likely also for this reason, some samples did not display TSG101, that is pat. #109, pat. #113 and pat. #114 (Fig. S3). Thus, results presented illustrate the presence of these exosome markers using SEC isolation of EVs from plasma of mUC patients but do not inform on the relative expression of CD9 or TSG101 among the samples.

Results from mice with xenografted tumours have suggested that there may be a correlation between numbers of EVs in plasma and the total tumour burden [44]. In cancer patients, the amount of EVs in plasma has not clearly been linked to disease burden [45, 46, 47]. However, an alteration in total and specific protein content of EVs has been found to correspond to disease progression in malignant melanoma patients [45, 46], while in head‐and‐neck cancer patients, alterations have been seen in EV protein cargo in response to therapy [47].

We analysed whether we could see a link between the level of EVs/ml plasma at baseline and PFS of the patients (Fig. 1D). Results showed that the concentration of EVs varied among the patients from 3.6 × 10^9^/ml to 6.1 × 10^11^/ml plasma but with no clear correlation to PFS (Pearson's correlation coefficient: 0.069; *P* ≥ 0.82). We also studied the change in EV levels in plasma during treatment (Fig. 1E). At day 8, two of the patients (pat. #113 and pat. #114), showed a two‐ to threefold increase in EV concentration relative to baseline, whereas in four patients, the amount of EVs was clearly reduced (pat. #101, pat. #106, pat. #102 and pat. #110) (Fig. 1E). At day 21, five patients displayed a twofold or higher amount of EVs relative to baseline (pat. #113, pat. #101, pat. #103, pat. #105 and pat. #112), while a reduced amount was found in pat. #102 (Fig. 1E). Thus, while there was a tendency to an increase in EVs at day 21 relative to baseline in the analysed samples, this observation should be interpretated with caution as our sample cohort is limited.

### 3.3 Protein profiling of EVs from mUC patient plasma reveals heterogeneity in protein expression signatures

**Fig. 2:**
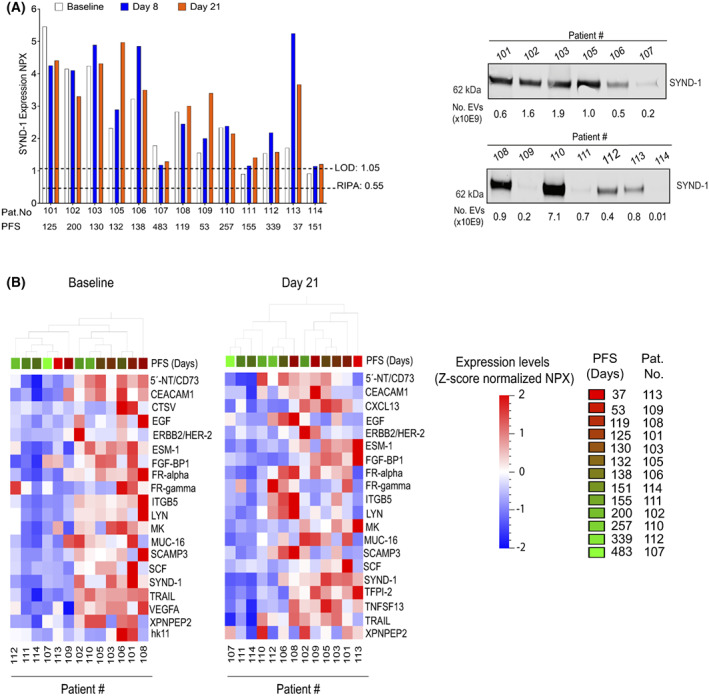
Protein profiling of extracellular vesicles from metastatic urothelial cancer patient plasma reveals heterogeneity in protein expression. Extracellular vesicles (EVs) isolated from plasma samples of metastatic urothelial cancer (mUC) patients at baseline and at day 8 or 21 were subject to proximity extension assay (PEA) protein profiling with the Oncology II^®^ assay. (A) *Left panel*: Expression [given as normalized protein expression (NPX) values] of SYND‐1 in EVs. No adjustment for the interpatient differences in the number of EVs analysed was made. The lower limit of detection (LOD) and the RIPA negative control levels are shown with a dotted line. The progression‐free survival (PFS) (in days) for the patients is stated. *Right panel*: Western blot profiling of SYND‐1 in EVs isolated from plasma samples at baseline. The number of EVs analysed in each sample is presented below the blot. The level of CD9 in the samples is shown in Fig. 1C. Please note that no normalisation for the number of EVs analysed was made. The presented result verify that SYND‐1 is expressed in the samples but does not describe its relative expression among the samples. (B) A selection of the proteins from the PEA analyses of the EVs with greatest variance in expression level among the samples is shown from baseline or at day 21. PFS of the patients in days is given. For description of the PEA data processing prior to this Qlucore analyses, please see Sections 2.4 and 2.5. Presented data in (A, B) are from one PEA profiling of one biological isolate of EVs from plasma of the individual patients.

For protein profiling, EVs isolated from the plasma samples of the mUC patients were subjected to PEA analyses on the Oncology II^®^ panel (see Section 2.4, Table S1). PEA analytics has previously been applied on tumour and plasma samples to reveal protein signatures for BM purpose [48, 49, 50, 51], as well as for protein profiling of EVs [40, 41]. When the EV samples were analysed by PEA, 86 of the 92 proteins in the PEA panel were found to be expressed over RIPA negative control in at least 50% of the analysed samples at baseline, and these markers were taken further into Qlucore bioinformatic analyses (Table S1). Among the proteins that demonstrated clear expression in the EVs was syndecan‐1 (SYND‐1) (Fig. 2A, *left panel*). SYND‐1 is involved in exosome biogenesis [52, 53, 54] and has earlier been reported in EVs from tumour cell lines and in bodily fluids of cancer patients [26, 36, 38, 41, 55, 56, 57, 58, 59]. We also confirmed SYND‐1 expression in the EVs from the mUC patient plasma samples by western blotting (Fig. 2A, *right panel*). However, as we applied different amounts of EVs in this profiling, one cannot relate the expression level of SYND‐1 in the different samples to each other. Expression of CD73, also known as 5′‐nucleotidase (5′‐NT), was evident in most of the EV samples (Fig. S4). CD73 has previously been identified in EVs from UC and other cancer cell lines, as well as in liquid biopsies of patients with UC and other tumours [29, 38, 55].

To study the protein profiles of EVs in individual plasma samples of the mUC patients, the PEA data were analysed with the qlucore bioinformatic software. The samples displayed diverse protein expression patterns both at baseline and at day 21 with some samples expressing an increased number of the proteins in the Oncology II^®^ panel while others having a decreased amount of the markers at day 21 vs baseline (data not shown). To visualise the protein profiles in the EV samples, we focused on the proteins showing the largest variance among the samples (Fig. 2B). As seen, the entire EV sample cohort differed at baseline and day 21 with respect to some of the proteins within these profiles with some proteins showing a higher variance in baseline vs day 21 samples, respectively. Moreover, we observed a trend to clustering of the samples with respect to PFS at day 21.

### 3.4 Rank regression analysis of protein expression patterns in EVs identifies a protein signature related to progression‐free survival

**Fig. 3:**
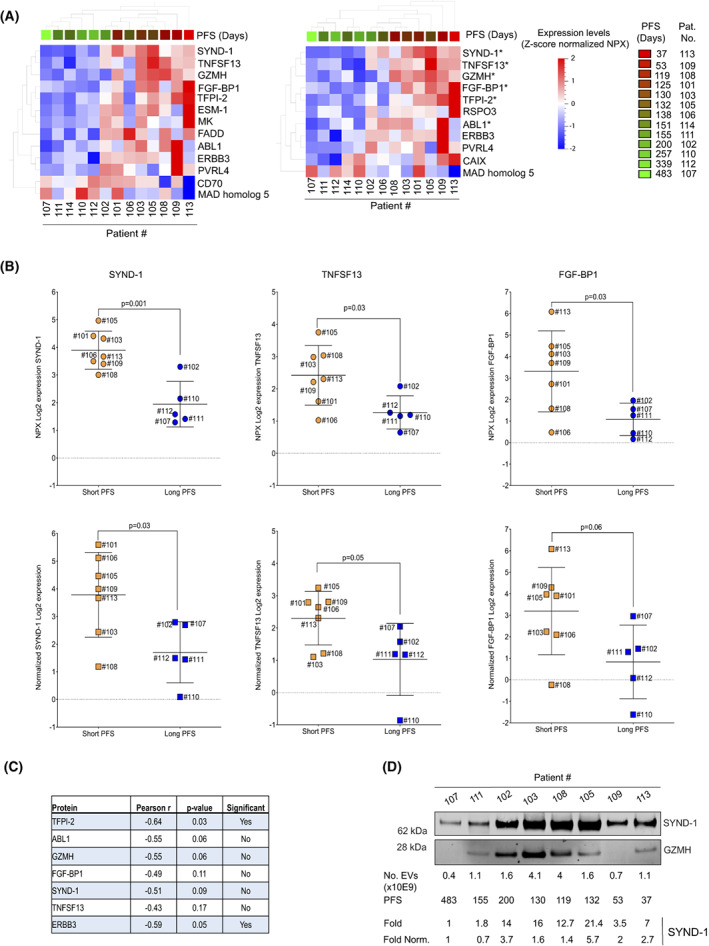
Protein profiling of extracellular vesicles in relation to progression‐free survival. Extracellular vesicles (EVs) isolated from plasma samples from metastatic urothelial cancer (mUC) patients at day 21 were subjected to proximity extension assay (PEA) protein profiling with the Oncology II^®^ assay (see Section 2.4). (A) Rank regression analyses of protein signatures in EVs related to progression‐free survival (PFS) [long (green) vs short (red)] of the study population. The qlucore software rank regression tool was used with *P* ≤ 0.05, and protein signatures were sorted without (*left panel*) or with (*right panel*) the number of EVs applied in the PEA profiling as elimination factor (see Section 2.5). For description of the processing of the PEA data prior to the Qlucore analyses with respect to samples showing protein expression below lower limit of detection (LOD) see Sections 2.4 and 2.5. The PEA data were also analysed with the XGBoost integrated tool of the qlucore software (see Section 2.5). The proteins sorted out by this approach and that also were revealed by univariate rank regression analyses are indicated with (*). (B) The normalized protein expression (NPX) values of individual proteins from (A) were analysed in EVs from plasma of patients with short (≤ 138 days) or long (> 138 days) PFS (*top panel*) or after normalising for the number of EVs used in the PEA profiling (*bottom panel*). Only proteins that were statistically significant using *t*‐test (see Section 2.5) at *P* ≤ 0.05 and ≤ 0.06 are shown. Bars represent standard deviation (SD) values. Proteins that only showed significant association with PFS when non‐normalised PEA data were used are presented in Fig. S5. Please note that pat. #114 was excluded in these analyses. For LOD of the proteins, see Table S1. (C) The expression of individual proteins from the PEA analyses was plotted with linearised values against PFS in linear regression analyses (see Section 2.5). The table indicates the Pearson correlation coefficient alongside *P*‐value for non‐normalised samples. Please, note that pat. #114 was excluded in these analyses. Presented data in A–C are from one PEA profiling of one biological isolate of EVs from plasma of the individual patients. (D) Western blot profiling of SYND‐1 and GZMH in EVs isolated from plasma samples of mUC patients at day 21. SYND‐1 and GZMH were profiled without normalising for the amount of EVs in the different samples. The fold expression of SYND‐1 relative to pat. #107 is given with (fold norm) or without (fold) normalisation for the number of EVs analysed. The densitometric quantification of SYND‐1 (without normalisation) is presented in Fig. S6. Data shown are from one western blot analysis of one biological isolate of EVs from the individual plasma samples of the patients.

To sort out plasma EV protein expression patterns that were linked to PFS, a rank regression univariate analysis was performed, which generated a protein signature in EVs at day 21, while at baseline or day 8, no signature was evident at the same statistical cut‐off (Fig. 3A, left panel). As the number of EVs applied in the PEA profiling could potentially influence some of the proteins identified in the signature, an adjustment of the signature was made using the number of EVs analysed as an elimination factor in the rank regression analysis (see Section 2.5). This generated a signature (Fig. 3A, right panel) consisting of SYND‐1, tumour necrosis factor ligand superfamily member 13 (TNFSF13), granzyme H (GZMH), fibroblast growth factor‐binding protein 1 (FGF‐BP1), tissue factor pathway inhibitor 2 (TFPI‐2), R‐spondin‐3 (RSPO3), tyrosine‐protein kinase ABL1 (ABL1), Erb‐b3 receptor tyrosine kinase 3 (ERBB3), poliovirus receptor‐related 4 (PVRL4)/nectin‐4, carbonic anhydrase IX (CAIX)/carbonic anhydrase 9 (CA9) and mothers against decapentaplegic homolog 5 (MAD homolog 5). The expression level of these different proteins varied among the EV samples from the mUC patient plasma samples with some of the proteins having a clear, over LOD, expression in almost all samples, that is SYND‐1, TNFSF13, FGF‐BP1 and TFPI‐2 while others, for example GZMH, ABL1, RSPO3, ERBB3, PVRL4/Nectin‐4 and MAD homolog 5, were below LOD but over RIPA negative control in the majority of the samples (for LOD and RIPA negative control values for each PEA reaction, see Table S1). In addition, a combined signature (i.e. classifier model) showing 77% accuracy was identified using a machine learning tool, XGBoost integrated within the qlucore software (see Section 2.5). This signature included primarily GZMH and SYND‐1 (both showed the highest significance), but also TNFSF13, FGF‐BP1, TFPI‐2, ABL1, FASLG, FADD and FR‐alpha. Some of these were indeed identified in univariate analyses, that is GZMH, SYND‐1, TNFSF13, FGF‐BP1, TFPI‐2 and ABL1 (Fig. 3A, right panel), further suggesting that these proteins expressed in EVs isolated from plasma were putatively associated with PFS of the mUC patients.

We also analysed the individual proteins within the PFS‐associated protein signature and their difference in expression levels in EVs from the mUC patient plasma samples comparing patients with long vs short PFS (Fig. 3B, Fig. S5). Among the proteins identified in the signature (Fig. 3A), SYND‐1, TNFSF13, FGF‐BP1, TFPI‐2, GZMH, ABL1 and ERBB3 all showed clear difference between the groups when analysed as individual proteins (Fig. 3B, top panel; Fig. S5). Moreover, a higher expression of SYND‐1, TNFSF13, and FGF‐BP1 in EVs from patients with short PFS was also significant (*P* ≤ 0.05, ≤ 0.06) when the number of EVs analysed was used to normalise the PEA data (Fig. 3B, lower panel). The association between these proteins and PFS was moreover analysed by linear regression analyses (Fig. 3C). Here, only high TFPI‐2 or ERBB3 levels were statistically associated with short PFS, while there was a tendency for ABL1 and GZMH when non‐normalised PEA data were used. None of the proteins were linked to PFS when the EV amount normalised PEA data was analysed.

We next analysed SYND‐1, GZMH and TFPI‐2 expression in EVs from mUC patient plasma samples taken at day 21 using western blot. For TFPI‐2, we failed to detect it in any EV sample likely because western blot is less sensitive than PEA. With respect to SYND‐1 and GZMH, we found that these proteins were expressed in the EV samples from plasma of mUC patients (Fig. 3D, top panel). As expected, a heterogeneous expression was seen among the samples from patients with long vs short PFS yet with some of the samples from patients with short PFS showing a higher SYND‐1 expression (Fig. 3D, Fig. S6). Similarly, we observed heterogeneous expression levels of GZMH among the samples; however, we cannot at this point clearly relate it to PFS. Thus, further analyses of the PFS‐associated protein signature of EVs are required using more sensitive techniques than western blot to validate our PEA findings.

### 3.5 PEA profiling of mUC plasma EV proteins to reveal signatures potentially associated with treatment response

**Fig. 4:**
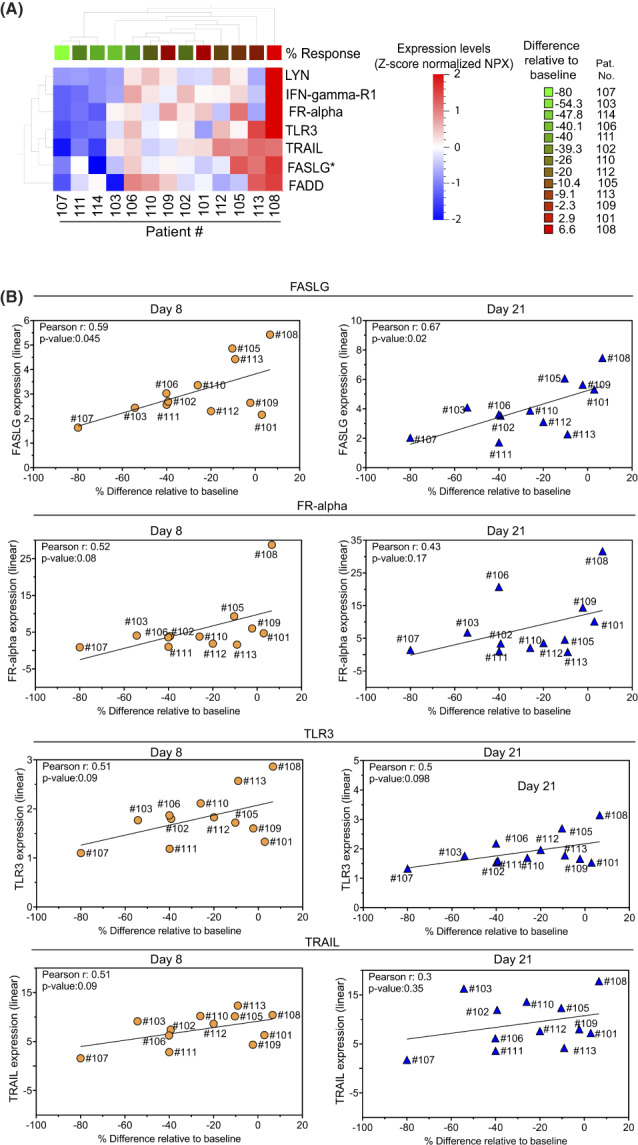
Profiling of extracellular vesicles to reveal putative protein signatures in relation to treatment response. Extracellular vesicles (EVs) isolated from plasma samples from metastatic urothelial cancer (mUC) patients at day 8 post‐treatment were subjected to proximity extension assay (PEA) protein profiling with the Oncology II^®^ assay as described in Section 2.4. Please note that for pat. #110, fractions 7–10 were analysed, while for all the other patients, fractions 6–10 were examined. (A) Rank regression analyses of protein signatures (*P* ≤ 0.05) in EVs related to best response of the patients evaluated by computerised tomography (CT) (Fig. S1B) are presented. The analyses were carried out as in Fig. 3A with the number of EVs analysed applied as an elimination factor (see Section 2.5). The PEA data were also analysed with the XGBoost integrated tool of the qlucore software (see Section 2.5) at day 21. Star (*) indicates that FASLG, which was revealed by univariate analyses, also was identified with this method. (B) The linear expression of indicated proteins in individual EV samples at day 8 and 21 was plotted against best CT response without normalisation for the number of EVs analysed. The line indicates results from the linear regression analyses. The Pearson correlation coefficient is given alongside the *P*‐value. The lower limit of detection (LOD) and RIPA negative control values are presented in Table S1. Please note that pat. #114 was excluded in these analyses (see Section 2.5).

Next, we explored whether alterations in the protein cargo of EVs showed an association with treatment response or refractoriness of the mUC patients (Fig. 4A). In EV samples from day 8, a protein signature was identified, which related to best treatment response as evaluated by CT when the number of EVs studied was used as an elimination factor. The signature, which was statistically significant, consisted of tyrosine‐protein kinase Lyn (LYN), IFN‐gamma‐R1 (IFNGR1), folate receptor alpha (FR‐alpha), Toll‐like receptor 3 (TLR3), TNF‐related apoptosis‐inducing ligand (TRAIL), FAS ligand (FASLG) and FAS‐associated protein with death domain (FADD). Some of these proteins were also present in a signature when no elimination with respect to amount of EVs analysed was done, that is LYN, IFNGR1, FR‐alpha, FASLG and FADD (data not shown). The proteins in the signature differed with respect to expression over LOD where LYN, FR‐alpha and TRAIL were found in almost all samples, while TLR3, FASLG, FADD and IFNGR1 had a lower expression in some samples still over negative RIPA control. Moreover, using XGBoost (see Section 2.5), a combined signature showing 82% accuracy was identified at day 21 and this signature included GZMH, FASLG and TFPI‐2. FASLG was also revealed by univariate analysis to be statistically significant for best treatment response (Fig. 4A). Of note, GZMH and TFPI‐2, which were identified by this XGBoost signature, were indeed found to be markers associated with PFS in both univariate and XGBoost analyses (Fig. 3A).

The correlation of some individual proteins in the best treatment response signature is presented at day 8 and 21 in Fig. 4B. FASLG expression was linked to best treatment response with a significant Pearson correlation coefficient value at both time points. FR‐alpha, TLR3 and TRAIL showed a tendency to association at day 8, while at day 21, only TLR3 was to some extent linked to best treatment response. When day 8 samples were normalised for the number of EVs applied in the PEA analyses, the Pearson correlation coefficient reached significance for FR‐alpha, while the other proteins did not show any statistically significant association (data not shown). In summary, our data suggest that profiling of proteins in EVs during the initial course of treatment can putatively reveal signatures related to treatment response where FASLG showed the strongest association. Moreover, using a machine learning classification approach, further proteins within EVs, some which overlapped with the PFS‐associated EV protein signature, were evident. Thus, results indicate a potential for early treatment assessment using EV protein profiling followed by bioinformatics. However, further validation of the individual proteins in the signature with alternative methods and in larger data sets are required.

## 4 Discussion

For platinum‐treatment refractory mUC patients, the treatment possibilities are limited, and the prognosis is poor. Moreover, the response to targeted and immune therapy is heterogeneous both among patients and in different phases of the disease [4, 9, 10, 60, 61, 62]. This calls for early treatment response monitoring methods, which can inform on putative treatment alternatives and allow for personalised treatment approaches.

To assess the tumour disease with liquid biopsy, for example, blood is appealing not only as sampling can be repeated during the treatment course but also as it can capture tumour disease heterogeneity. In this exploratory study on protein profiling of EVs isolated from plasma of mUC patients, we show feasibility to reveal protein signatures in EVs that associate with PFS (i.e. SYND‐1, TNFSF13, FGF‐BP1, TFPI‐2, GZMH, ABL1 and ERBB3). Our analyses also put forward some putative proteins associated with treatment response including FR‐alpha, TLR 3, TRAIL and FASLG.

We found that EVs from plasma were of sEV size [23], and western blot analyses demonstrated expression of CD9 and TSG101, both markers of exosomes [23]. We also verified expression of SYND‐1 in EVs, a protein earlier described in EVs isolated from cancer samples, for example from cell culture media of cancer cell lines or in liquid biopsies including plasma of cancer patients with different tumour types [26, 36, 38, 41, 52, 54, 56, 57, 58, 59]. A heterogeneity in the size of the EVs was evident both at baseline and after treatment in our cohort. The cellular origin of the EVs, for example tumour vs nontumour cells, can likely in part explain the observed diverse EV sizes, but we cannot rule out that the use of different SEC fractions for some of the samples may have impacted on the result. It is, however, unlikely given that the size of the EVs in the samples that were isolated using different SEC fractions did not diverge more over the different time points than other samples in which SEC fractions 6–10 were pooled and analysed. Nevertheless, it is preferred that the same SEC fractions are used when isolating EVs from different samples as it otherwise may introduce bias. In the PEA profiling and western blot analyses, all samples except pat. #110 at day 8 were pooled from SEC fractions 6–10, concentrated and analysed. Albeit the divergent pat. #110 sample used (isolated from pooled SEC fraction 7–10) could potentially have influenced the identification of the best treatment response signature at day 8, we cannot see that pat. #110 is an outlier in the presented data.

We observed a range of EVs/ml plasma in baseline samples, between ~ 3.6 × 10^9^ and ~ 6.1 × 10^11^ per ml of plasma. These levels are in line with previous reports from plasma of malignant melanoma patients [63]. One could speculate that the amount of EVs should be higher in the patients with shorter PFS as this may be associated with a larger tumour burden as reported from xenograft studies in mice [44]. We could, however, not confirm this in our study. Possible explanations are not only that tumours display a variation in intra‐ and interindividual pharmacodynamic effect on EVs released at days 8 and 21 but also that patients may be heterogeneous when it comes to clearance of EVs from circulation as earlier revealed in tumour studies in mice [44].

The lack of association between EVs/ml of plasma and PFS in our study is in line with reports from other tumour types. Thus, Peinado et al. did not reveal any association between the amounts of EVs/exosomes in plasma of malignant melanoma patients and tumour stage, but it was demonstrated that protein amount was higher in exosomes from patients with stage IV disease [46]. Conversely, if patients with stage IV disease had low protein content in their EVs, a better outcome was seen [46]. Unfortunately, our protein concentration measurements did not give consistent results. Hence, we cannot conclude whether protein amount of EVs can be linked to PFS in our patient cohort as reported both in plasma from head‐and‐neck cancer patients after photodynamic therapy [47] and in exudative seroma from melanoma patients after lymphadenectomy [45].

We would like to emphasise that multiplex protein profiling of EVs in relation to mUC treatment response is a rather nonexplored area. Moreover, our study is exploratory in its nature, performed on a rather small cohort of patients with heterogeneous distribution of metastases. We also used different amounts of EVs for the PEA protein profiling of the different patient plasma samples, which could have influenced the observed EV protein profiles. However, as the PEA data processing to generate PFS or best response signatures included elimination of EV amount variations in the PEA profiling step, this is to at least some extent compensated for. How to normalise protein expression data with respect to the amount of EVs profiled is still not clear as tumour‐derived EVs can be low in a sample with high amount of EVs/ml plasma and vice versa. Some researchers use protein/EV amount in a sample to address this issue, which unfortunately was not feasible in the current study as described above. An alternative approach that could have been taken would have been to ‘fish out’ EVs of epithelial origin from the bulk of the EVs in the plasma samples (assuming that tumour EVs express EpCAM to a high degree) and subsequently use this amount of EVs for normalisation of the samples. Such an approach could be feasible using EpCAM‐magnetic beads, for example. We nevertheless would like to stress that in our results in which a putative PFS‐associated protein signature was revealed, we do see clear overlap among the proteins in the signatures with and without normalisation of the EV input to the PEA analyses. So even with our crude method, we likely capture protein signatures related to tumour possibly because the used PEA panel to a large degree consists of cancer‐related proteins. We observed that EV samples from some patients displayed low expression of proteins in some but not all of the presented PEA results, for example pat. #114 and pat. #107. It may of course be related to the fact that we used different amount of EVs in the analyses, but it may also be a result of the molecular characteristics of the individual patient plasma EV subsets studied, for example differences in the proportion of tumour‐derived EVs of the total EVs in the plasma sample analysed, as well as the clinical response of the patient to the given treatment. For example, the EV sample from pat. #107, which displayed low levels of all proteins in the PEA panel in several of the analyses, was obtained from a patient who had a very good PFS and treatment response (Table 1). Hence, one would assume that the cancer‐associated proteins in the Oncology II panel should have a low expression in at least the tumour‐derived EVs from that patient plasma sample.

We identified a protein signature consisting of SYND‐1, TNFSF13, FGF‐BP1, TFPI‐2, GZMH, ABL1 and ERBB3 to be putatively associated with PFS. Moreover, in a machine learning method for classification (i.e. XGBoost), GZMH, SYND1, TNFSF13, FGF‐BP1, TFPI‐2 and ABL1 were also found to be associated with PFS further strengthening the data. We also studied the individual proteins in the PFS‐associated protein signature in relation to long vs short PFS of the patients, and here, we observed that SYND‐1, TNFSF13 and FGF‐BP1 were also significant when normalising the PEA data with respect to the amounts of EVs analysed by PEA, while the other proteins, for example TFPI‐2, GZMH, ABL1 and ERBB3, were not. As it previously has been shown that amount of EVs is not always correlated to clinical parameters [45, 46, 47] and given that it is reported that some of EVs in plasma of cancer patients can be enriched in protein amount (which links to clinical stage and/or metastasis [45, 46] and treatment response [47]), one could question whether normalising for the number of EVs analysed is a valid approach. From our data, we can see that some protein markers then are lost with respect to clinical parameter association. We also would like to stress that as we only verified SYND‐1 and GZMH to be expressed in EVs by western blot. This is likely due to the method not being sensitive enough. Thus, both expanded analyses of the PFS signature in other mUC patient plasma cohorts and further validation of individual markers within the signature with more sensitive methods are required to take our EV protein profiling results towards clinical implementation.

We explored whether the proteins with potential association with PFS previously have been reported in EVs/exosomes from tumour cells and/or in body fluids of cancer patients using Vesiclepedia (http://www.microvesicles.org/index.html) [64] and other public sources. SYND‐1 [25, 26, 36, 38, 41, 52, 54, 56, 57, 58, 59], TFPI‐2, [26, 56, 58], FGF‐BP1 [56, 58, 59] and ABL1 [26] have indeed been revealed in cancer EVs/exosomes or in liquid biopsies of cancer patients using primarily MS‐based analyses. We failed to found reports on GZMH and TNFSF13 expression in EVs of cancer cells. A possible explanation could be that we here used PEA, which is an antibody‐based assay with high sensitivity, while most of the above‐mentioned articles applied MS‐based analyses. In fact, even by profiling EVs with PEA, GZMH expression was only seen over LOD in a fraction of the samples, suggesting that it is a low abundant protein in EVs, at least in these mUC patient plasma samples.

One of the proteins in EVs, which showed a tendency to association with short PFS of the mUC patients, was SYND‐1, a protein expressed in multiple different tumour types [65, 66]. SYND‐1 may, via its heparan sulphate (HS) chains, interact with cytokines, chemokines, lipid metabolism, growth factors and extracellular matrix components [53, 65]. Moreover, SYND‐1 also regulate exosome formation and release via multiple proteins, for example CD63, TSG101, RAB7, ARF6, and heparanase [52, 53, 54]. It has been demonstrated that SYND‐1 may regulate UC *in vitro* cell viability and *in vivo* in mice when tumours are grown orthotopically [67]. A higher SYND‐1 expression was also evident in advanced UC tumours with invasive growth, while a less expression was found in low‐grade tumours [67]. Importantly, SYND‐1 positivity was linked to UC recurrence after resection. In a study of tumours from UC patients with different stages, Szarvas et al. [68] reported that SYND‐1 membrane positivity in the tumour cells decreased as tumour stage increased with the lowest level observed in metastatic cases but with a concomitant increase in expression in tumour stroma. Interestingly, when SYND‐1 ectodomain was measured in serum from the same UC patient cohort, a higher level was evident in patients with muscle‐invasive UC as compared to noncancer cases or cases with non‐muscle‐invasive UC [68]. Moreover, high SYND‐1 pretreatment serum levels and presence of distant metastases were recently demonstrated to be risk factors for poor OS [69]. Our finding that SYND‐1 expression in EVs shows a tendency to link to short PFS of metastatic UC is in line with these data.

With respect to SYND‐1, two studies reported on a BM potential in EVs isolated from plasma and urine of cancer patients, respectively [36, 41]. Thus, the Belting‐ team showed that SYND‐1 in plasma extracellular vesicles (plEVs) could sort out high‐grade glioblastoma multiforme from low‐grade glioma [41]. plEV‐expressed SYND‐1 was also linked to tumour SYND‐1 expression levels. Tomiyama et al. identified SYND‐1 in an extensive protein profiling study on EVs isolated from urine and tissue explants of UC patients. In that study, SYND‐1 was moreover selected as one BM candidate out of about 20 and used in a selected reaction monitoring/multiple reaction monitoring (SRM/MRM) approach [36]. Results showed that SYND‐1 had at least a twofold higher expression level in EVs from urine of UC patients relative to healthy controls, thus confirming a BM potential.

In our study, one of the proteins of EVs, which showed association with best treatment response, was FR‐alpha, a plasma membrane protein with affinity for folate and which has been evaluated as a therapeutic target for some epithelial tumour types [70]. FR‐alpha has previously been found in EVs from different tumours [56, 58] and in urine from UC or prostate cancer patients [26, 27, 29]. Thus, Hiltbrunner *et al*., with the aim to identify putative BMs in EVs that could inform on tumour left behind cystectomy, found that FR‐alpha was among a 40‐protein large subset that had a higher expression in EVs isolated from urine of the bladder of UC patients compared to ditto from ureter urine of UC patients [29]. It was suggested that FR‐alpha and the other proteins identified to have a higher expression in EVs from bladder urine after surgery should be further explored for BM purpose in terms of assessing remaining tumour disease.

Another interesting issue is whether protein cargo of EVs isolated from plasma can be linked to tumour localisation or metastatic site. A large number of studies have demonstrated that EVs from tumours, for example malignant melanoma, lung, prostate and pancreatic cancer, promote the metastatic niche and that the EV cargo holds specific messages allowing tumours to be established in certain tissues [46, 71, 72, 73, 74, 75]. In our limited patient cohort, consisting of EV plasma samples from mUC patients mainly with a primary tumour in the bladder, we cannot address whether primary UC tumour localisation is associated with certain EV protein profiles. Moreover, the EVs that we analysed are from plasma of metastatic UC patients and most of these patients have more than one organ affected by metastases, which may secrete EVs, making such analyses further complicated. In mUC patients, it is clinically relevant to discuss whether one can see differences in protein profiles of EVs when patients have visceral metastasis or not as the visceral metastasis influence the prognosis of the disease [76]. We accordingly looked for protein signatures in EVs that may be different between these two patient groups; however, as our cohort was rather small, we only observed four proteins, that is WIF‐1, CXCL17, LYPD3 and GZMB, that showed association yet with high probability of false positivity. Nevertheless, further studies on association of EV protein profiles in relation to metastatic localisation are warranted in larger mUC patient plasma cohorts.

There is a great attention to use EVs isolated from urine for BM discovery and cancer disease monitoring purpose in prostate, renal, UC and other cancer malignancies [27, 28, 77]. Chen et al. [25] reported on urine EV proteome profiling from patients with low‐ and high‐grade UC, as well as Hernia. A differential expression of APOA1, CD5L, FGA, FGB, FGG, HPR and HP was found in the urine‐derived microparticles from patients with low‐ or high‐grade UC. Dhondt et al. [26] did an in‐depth study of the proteome of EVs isolated not only from urine primarily from prostate cancer patients but also from some patients with UC and renal cancer. Results showed that some of the proteins found in EVs were shared among these cancer types, while others were tumour‐specific, and with respect to UC, the EVs expressed high levels of UPK1A, UPK1B, UPK2 and UPK3B. Unfortunately, the proteins that we found in our study to be associated with PFS or best treatment response were not visible in the proteomic data of EVs isolated from patients with UC [26] or from data pointed out by Chen et al. [25]. Of course, this may be related to the fact that we analysed EVs isolated from plasma, which clearly differ from EVs isolated from urine. It may also be explained by the fact that all our samples were isolated from advanced cancer patients, while the studies on EVs from urine of UC patients indicated above included also early‐stage disease. Given the above‐mentioned reports on SYND‐1 [36] and FR‐alpha in EVs isolated from urine of UC patients [29], which demonstrated BM potential, it may be relevant to study those proteins in EVs from urine also of mUC patients in relation to clinical outcome. However, analyses of EVs isolated from urine are still more relevant for early disease with a tumour confined to the bladder rather than in a metastatic setting where the patient's tumour burden is spread also outside the bladder or the urinary system to multiple organs. Thus, albeit EVs isolated from urine are attractive as a noninvasive source of BMs, further studies are required to address whether such EVs also can be used for BM purpose in advanced mUC patients.

In this study, we used PEA analytics followed by univariate analysis to put forward a putative protein signature in EVs consisting of SYND‐1, TNFSF13, FGF‐BP1, TFPI‐2, GZMH, ABL1 and ERBB3, which was associated with PFS. All these markers, except for ERBB3, were also confirmed using a multivariate approach, that is a machine learning classification algorithm (XGBoost), which provide further support for these proteins to be linked to PFS. We also found an EV protein signature by univariate analysis, which, at day 8, was to a certain degree associated with best treatment response, while at day 21, we did not identify such a signature. The day 8 signature comprised of multiple proteins with FR‐alpha, TLR3, TRAIL and FASLG showing some independent correlation to treatment response albeit without all demonstrating statistical significance likely because of the small size of the present cohort. At first, it may be puzzling that this EV signature is different from the one associated with PFS. However, a signature associated with PFS is likely driven by a combination of intrinsic tumour properties such as proliferation capacity, metastatic status, overall tumour burden and sensitivity to the given treatment, while best treatment response primarily is related to the latter. Of note, applying XGBoost analyses on the PEA profiling data obtained from the EV samples revealed GZMH, FASLG, TFPI‐2, FR‐alpha and FADD to be mutual between PFS and best treatment response outcome. Together with SYND‐1, TNFSF13 and FGF‐BP1, we suggest that these proteins should be nominated for further validation as possible BMs of plasma‐isolated EVs of mUC patients.

## 5 Conclusions

In this exploratory study, we report that protein profiling of EVs isolated from plasma of mUC patients can identify protein signatures associated with PFS and treatment response. Our findings illustrate that profiling of EV protein cargo may hold potential as source of prognostic and/or predictive BMs for mUC and warrant further studies in extended patient cohorts and with focus on tumour‐derived EVs.

## Author contributions

KV, AU, PH, C‐HS, PS, BF and RL conceptualised and designed the study. KV, BF, PH, C‐HS, VA and AU developed the methodology. PH, VA, BF, C‐HS, KH, KV and AU acquired the data. KV, AU, PH, BF, C‐HS, VA, KH and RL analysed and interpreted the data. KV, AU, PH, BF and C‐HS wrote the manuscript. AU and KV supervised the study. All authors reviewed the manuscript.

## Conflict of interest

Per Sandstrom is an employee of Bayer US. Anders Ullén received support for the Vinsor study from Bayer AB and Pierre‐Fabre AB. The other authors have no disclosure related to the work.

## Supporting information


**Fig. S1.** Study outline and tumour response in a subset of metastatic urothelial cancer (mUC) patients from the Vinsor trial.
**Fig. S2.** Nanoparticle tracking analysis of extracellular vesicles.
**Fig. S3.** Western blot profiling of extracellular vesicles from metastatic urothelial cancer (mUC) patient plasma at baseline.
**Fig. S4.** Expression of CD73/5′‐nucleotidase (5′‐NT) in extracellular vesicles isolated from metastatic urothelial cancer (mUC) patient plasma.
**Fig. S5.** Proteins in extracellular vesicles at day 21 associated with progression‐free survival (PFS).
**Fig. S6.** SYND‐1 expression in extracellular vesicles at Day 21.
**Table S1.** List of the proteins included in the proximity extension assay (PEA) on the Oncology II^®^ panel applied for profiling of extracellular vesicles (EVs) from plasma of metastatic urothelial cancer (mUC) patients.
**Table S2.** Localisation of the primary urothelial carcinoma and metastases of the analysed patient cohort.

## Data Availability

Data supporting the findings are reported in the Supplementary material. Data not included in the Supporting Information are available from the corresponding authors (kristina.viktorsson@ki.se; or anders.ullen@regionstockholm.se) upon request with explanation for use.
