# Supplementary material for: Profiling of extracellular vesicles of metastatic urothelial cancer patients to discover protein signatures related to treatment outcome
Source: Mol Oncol. 2022 Aug 12;16(20):3620–41. doi: 10.1002/1878-0261.13288 (PMC9580890; doi:10.1002/1878-0261.13288)
Supplement: Supplementary file 1 — Fig. S1. Study outline and tumour response in a subset of metastatic urothelial cancer (mUC) patients from the Vinsor trial. Fig. S2. Nanoparticle tracking analysis of extracellular vesicles. Fig. S3. Western blot profiling of extracellular vesicles from metastatic urothelial cancer (mUC) patient plasma at baseline. Fig. S4. Expression of CD73/5′‐nucleotidase (5′‐NT) in extracellular vesicles isolated from metastatic urothelial cancer (mUC) patient plasma. Fig. S5. Proteins in extracellular vesicles at day 21 associated with progression‐free survival (PFS). Fig. S6. SYND‐1 expression in extracellular vesicles at Day 21. Table S1. List of the proteins included in the proximity extension assay (PEA) on the Oncology II® panel applied for profiling of extracellular vesicles (EVs) from plasma of metastatic urothelial cancer (mUC) patients. Table S2. Localisation of the primary urothelial carcinoma and metastases of the analysed patient cohort. [file MOL2-16-3620-s001.pdf]

Supplementary Figures and Tables

Profiling of extracellular vesicles of metastatic urothelial cancer patients to discover protein signatures related to treatment outcome

Kristina Viktorsson [1*], Petra Hååg[1], Carl-Henrik Shah[1,2], Bo Franzén[1], Vasiliki Arapi[1], Karin Holmsten[1,3] Per Sandström[1,2], Rolf Lewensohn[1,4], and Anders Ullén[1,2*]

[1] Department of Oncology-Pathology, Karolinska Institutet, SE-171 64 Solna, Sweden. [2]Department of Pelvic cancer, Genitourinary oncology and urology unit, Karolinska University Hospital, SE-171 64 Solna, Sweden. [3]Department of Oncology, Capio Sankt Göran's Hospital, SE-112 19 Stockholm, Sweden. [4]Theme Cancer, Medical Unit head and neck, lung, and skin tumors, Thoracic Oncology Center, Karolinska University Hospital, SE-171 64 Solna, Sweden.

Supplementary Table 1. List of the proteins included in the Proximity Extension Assay (PEA) on the Oncology II® panel applied for profiling of Extracellular Vesicles (EVs) from plasma of metastatic urothelial cancer (mUC) patients. The Lower Limit of Detection (LOD) and the RIPA negative control values of the different protein reactions in the analyses are given as Normalized Protein eXpression (NPX) values. The proteins excluded in the Qlucore bioinformatic analyses of the PEA profiling data of EVs are marked in italics.

No.	Protein	Uniprot ID	LOD (NPX)	RIPA (NPX)	Included Qlucore Bioinformatic analyses (Yes (Y)/No (N))
1	5'-NT/CD73	P21589	0,687	-0,319	Y
2	ABL1	P00519	0,260	-0,364	Y
3	ADAM 8	P78325	-1,187	-1,272	Y
4	ADAM-TS 15	Q8TE58	0,855	-0,420	Y
5	ANXA1	P04083	0,791	0,063	Y
6	AREG	P15514	0,966	0,154	Y
7	CAIX/ CA9	Q16790	-0,599	-0,980	Y
8	CD160	O95971	1,621	0,914	Y
9	CD27	P26842	1,373	0,934	Y
10	CD48	P09326	0,596	-0,446	Y
11	CD70	P32970	-0,095	-2,471	Y
12	***CD207***	***Q9UJ71***	***0,307***	***-0,176***	***N***
13	CDKN1A	P38936	-0,115	-0,778	Y
14	CEACAM1	P13688	0,711	0,066	Y
15	***CEACAM5***	***P06731***	***0,480***	***0,135***	***N***
16	CPE	P16870	1,810	0,438	Y
17	CRNN	Q9UBG3	-0,092	-1,108	Y
18	CTSV	O60911	2,062	1,247	Y
19	CXCL13	O43927	0,593	-0,489	Y
20	CXCL17	Q6UXB2	0,114	-0,245	Y
21	CYR61	O00622	1,739	0,640	Y
22	DLL1	O00548	-1,095	-1,880	Y
23	EGF	P01133	1,343	0,573	Y
24	EPHA2	P29317	0,330	-0,361	Y
25	ERBB2	P04626	1,532	0,552	Y
26	ERBB3	P21860	-0,418	-1,403	Y
27	ERBB4	Q15303	-1,106	-1,610	Y
28	ESM-1	Q9NQ30	1,292	0,412	Y
29	FADD	Q13158	0,769	-0,407	Y
30	FASLG	P48023	1,545	0,606	Y
31	***FCRLB***	***Q6BAA4***	***0,718***	***0,599***	***N***
32	FGF-BP1	Q14512	0,172	-0,910	Y
33	Folate receptor alpha/FR-α	P15328	-0,692	-0,577	Y
34	Folate receptor gamma/FR-γ	P41439	1,086	0,007	Y
35	FURIN	P09958	1,453	0,776	Y
36	Gal-1	P09382	0,488	-0,408	Y
37	***GPC1***	***P35052***	***0,703***	***0,345***	***N***
38	GPNMB	Q14956	1,385	0,481	Y
39	GZMB	P10144	0,387	-0,353	Y
40	GZMH	P20718	0,625	-0,043	Y
41	HGF	P14210	0,214	-0,353	Y
42	hK11	Q9UBX7	1,895	0,868	Y
43	hK14	Q9P0G3	1,928	1,491	Y
44	hK8	O60259	1,519	0,686	Y
45	ICOSLG	O75144	1,130	0,369	Y
46	IFN-gamma-R1	P15260	1,233	0,469	Y
47	IGF1R	P08069	0,480	-0,133	Y
48	IL6	P05231	1,547	0,390	Y
49	ITGAV	P06756	0,988	0,485	Y
50	ITGB5	P18084	1,854	0,607	Y
51	KLK13	Q9UKR3	0,701	-0,379	Y
52	LY9	Q9HBG7	0,155	-0,537	Y
53	LYN	P07948	0,308	0,244	Y
54	LYPD3	O95274	-0,762	-1,431	Y
55	MAD homolog 5	Q99717	0,443	-0,335	Y
56	MetAP 2	P50579	1,501	0,552	Y
57	MIA	Q16674	0,786	-0,282	Y

58	MIC-A/B	Q29983, Q29980	-0,129	-0,498	Y
59	MK/MDK	P21741	-0,144	-0,929	Y
60	MSLN	Q13421	0,629	-0,040	Y
61	MUC-16	Q8WXI7	1,258	0,703	Y
62	PODXL	O00592	0,682	-0,736	Y
63	PPY	P01298	0,429	-0,443	Y
64	PVRL4/Nectin-4	Q96NY8	1,784	0,720	Y
65	RET	P07949	0,231	-0,252	Y
66	RSPO3	Q9BXY4	0,516	-0,568	Y
67	***S100A4***	***P26447***	***-0,174***	***-0,731***	***N***
68	S100A11	P31949	1,245	0,004	Y
69	SCAMP3	O14828	0,225	-0,435	Y
70	SCF	P21583	0,584	-0,164	Y
71	SEZ6L	Q9BYH1	0,275	-0,826	Y
72	SPARC	P09486	0,477	-0,185	Y
73	SYND-1/SDC1	P18827	1,054	0,547	Y
74	TCL1A	P56279	0,492	-0,474	Y
75	TFPI-2	P48307	1,308	0,129	Y
76	TGF-alpha	P01135	-0,356	-1,151	Y
77	TGFR-2	P37173	1,312	0,339	Y
78	TLR3	O15455	1,221	0,394	Y
79	TNFRSF4	P43489	1,303	-0,908	Y
80	***TNFRSF19***	***Q9NS68***	***-0,013***	***0,069***	***N***
81	TNFRSF6B	O95407	1,438	0,302	Y
82	TNFSF13	O75888	-0,275	-0,958	Y
83	TRAIL	P50591	0,422	-0,120	Y
84	TXLNA	P40222	1,234	0,567	Y
85	VEGFA	P15692	0,817	-0,311	Y
86	VEGFR-2	P35968	1,009	-0,276	Y
87	VEGFR-3	P35916	1,032	-0,672	Y
88	VIM	P08670	-0,541	-1,166	Y
89	WFDC2	Q14508	1,228	0,195	Y
90	WIF-1	Q9Y5W5	1,132	0,562	Y
91	WISP-1	O95388	0,497	0,247	Y
92	XPNPEP2	O43895	1,207	-0,051	Y

Supplementary Table 2. Localisation of the primary urothelial carcinoma and metastases of the analysed patient cohort.

Patient No.	Primary urothelial carcinoma location	Metastatic lesion localization	Visceral metastasis[1] Yes/No
**101**	Bladder	Lymph nodes (multiple)	No
**102**	Bladder	Lymph nodes (multiple) and peritoneal carcinosis	Yes
**103**	Bladder	Lymph nodes (multiple)	No
**105**	Renal pelvis	Lymph node and adrenal gland	Yes
**106**	Ureter	Liver, bone and lymph nodes (multiple)	Yes
**107**	Bladder	Lymph nodes (multiple)	No
**108**	Bladder	Bone	Yes
**109**	Bladder	Lymph nodes (multiple)	No
**110**	Renal pelvis	Liver and bone	Yes
**111**	Bladder	Lungs and lymph nodes (multiple)	Yes
**112**	Bladder	Lung	Yes
**113**	Bladder	Bone, adrenal gland and lymph nodes (multiple)	Yes
**114**	Ureter	Lung and lymph nodes (multiple)	Yes

[1]Visceral metastases include metastases in liver, lung, bone, non-lymph node or soft tissue according to Bellmunt JCO 2010 Apr 10;28(11):1850-5.doi: 10.1200/JCO.2009.25.4599. Epub 2010 Mar 15.

Supplementary Figure 1

(A)

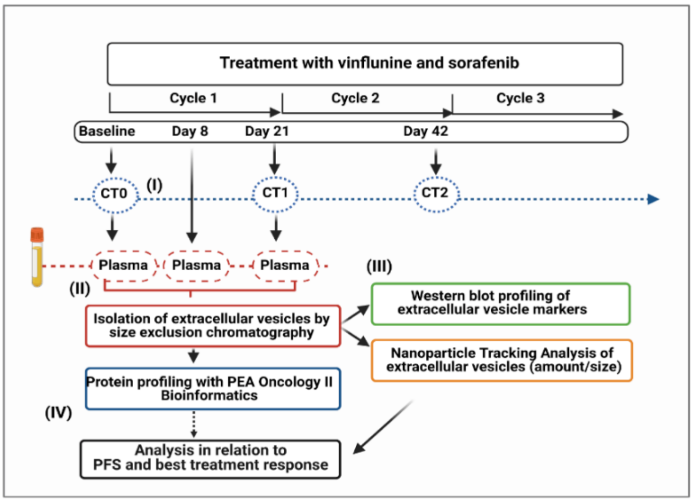

(B)

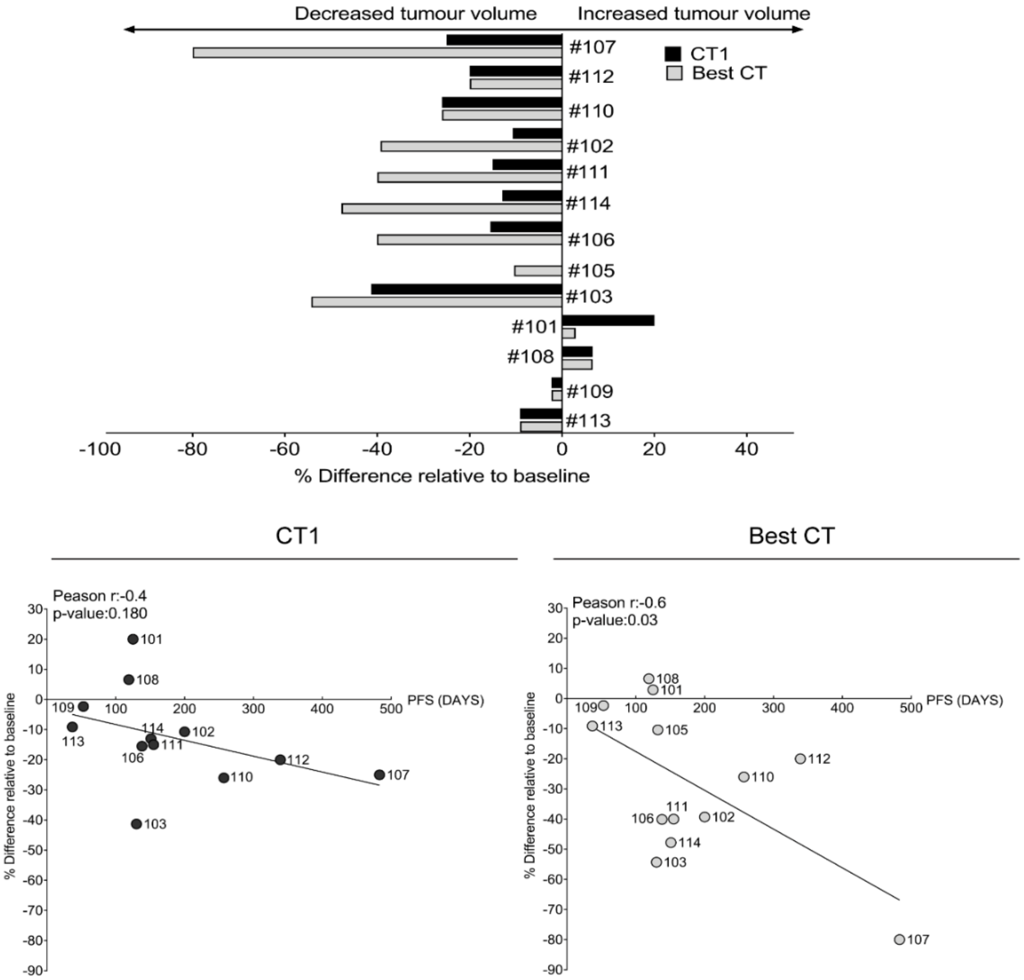

Supplementary Figure 1. Study outline and tumour response in a subset of metastatic urothelial cancer (mUC) patients from the Vinsor trial. (A). Study overview. (I). Computed tomography (CT) as a method for evaluation of tumour target lesion(s) and plasma samples

(n=13) were obtained at baseline (CT0), and at indicated days post study treatment initiation. CT scans were carried out prior to the first three treatment cycles and thereafter prior to every other cycle. (II). Size exclusion chromatography (SEC) was used for isolation of extracellular vesicles (EVs) from plasma. (III). EVs were profiled for size and concentration by Nanoparticle Tracking Analysis (NTA) and for expression of EV markers by Western blotting. (IV). EVs at baseline, day 8 and 21 were subject to Proximity Extension Assay (PEA) profiling with the Oncology II® assay. Bioinformatic exploration of data by Qlucore Omics Explorer 3.5 software was applied to sort out protein expression signatures related to Progression Free Survival (PFS) or best treatment response. (**B**). The percentage difference in the sum of tumour target lesions after one treatment cycle (CT1) and best response CT (within study) relative to baseline was calculated for each patient. *Top:* Data is presented for each patient stratified on their PFS in days. *Bottom*: % difference in the sum of tumour target lesions relative to baseline is given for each patient at CT1 and at best response in relation to PFS in days. Pearson coefficient was calculated to be r=-0.4 for CT1 vs. PFS (p=0.18, ns) and r=-0.6 for best response vs. PFS (p=0.03, significant).

Supplementary Figure 2

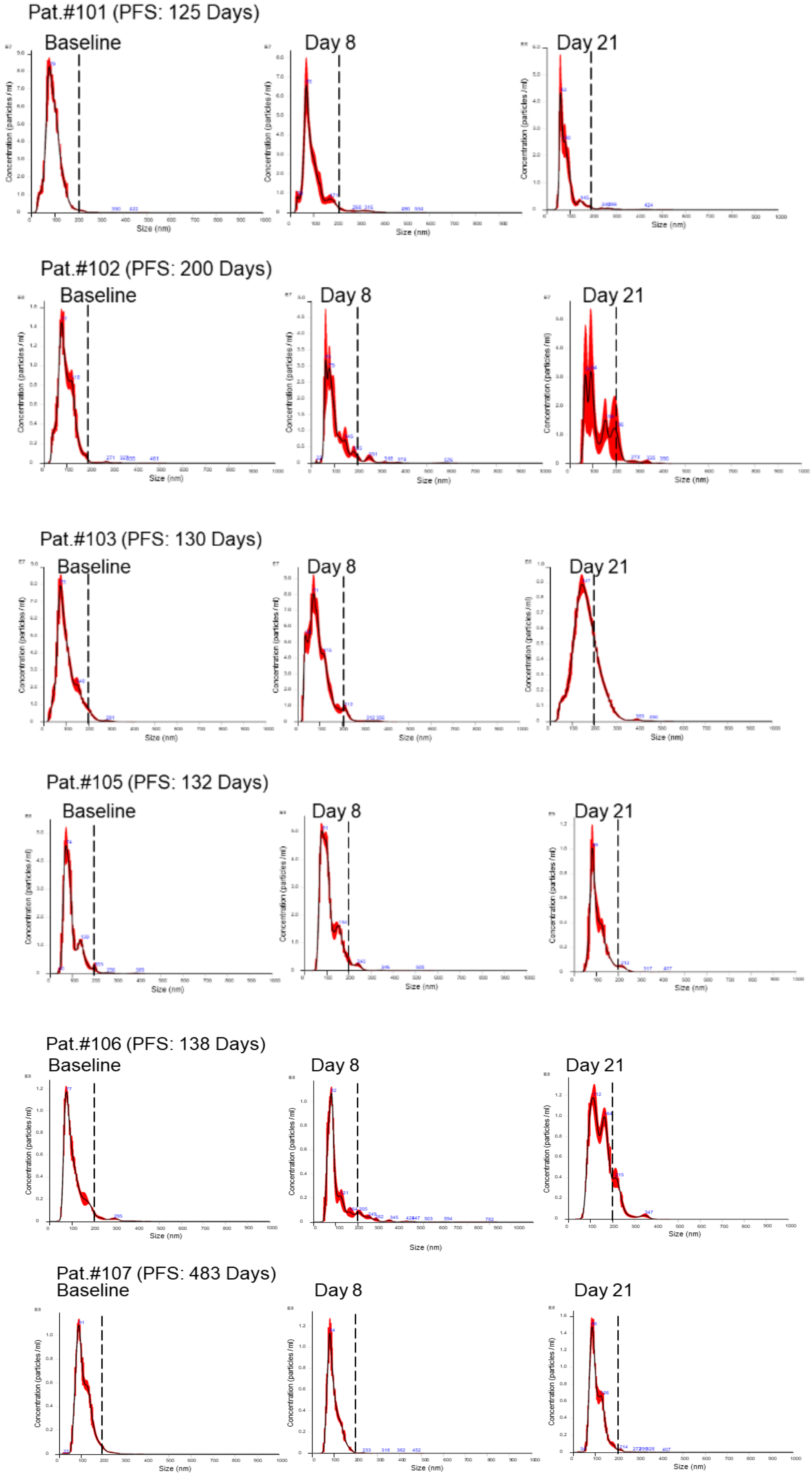

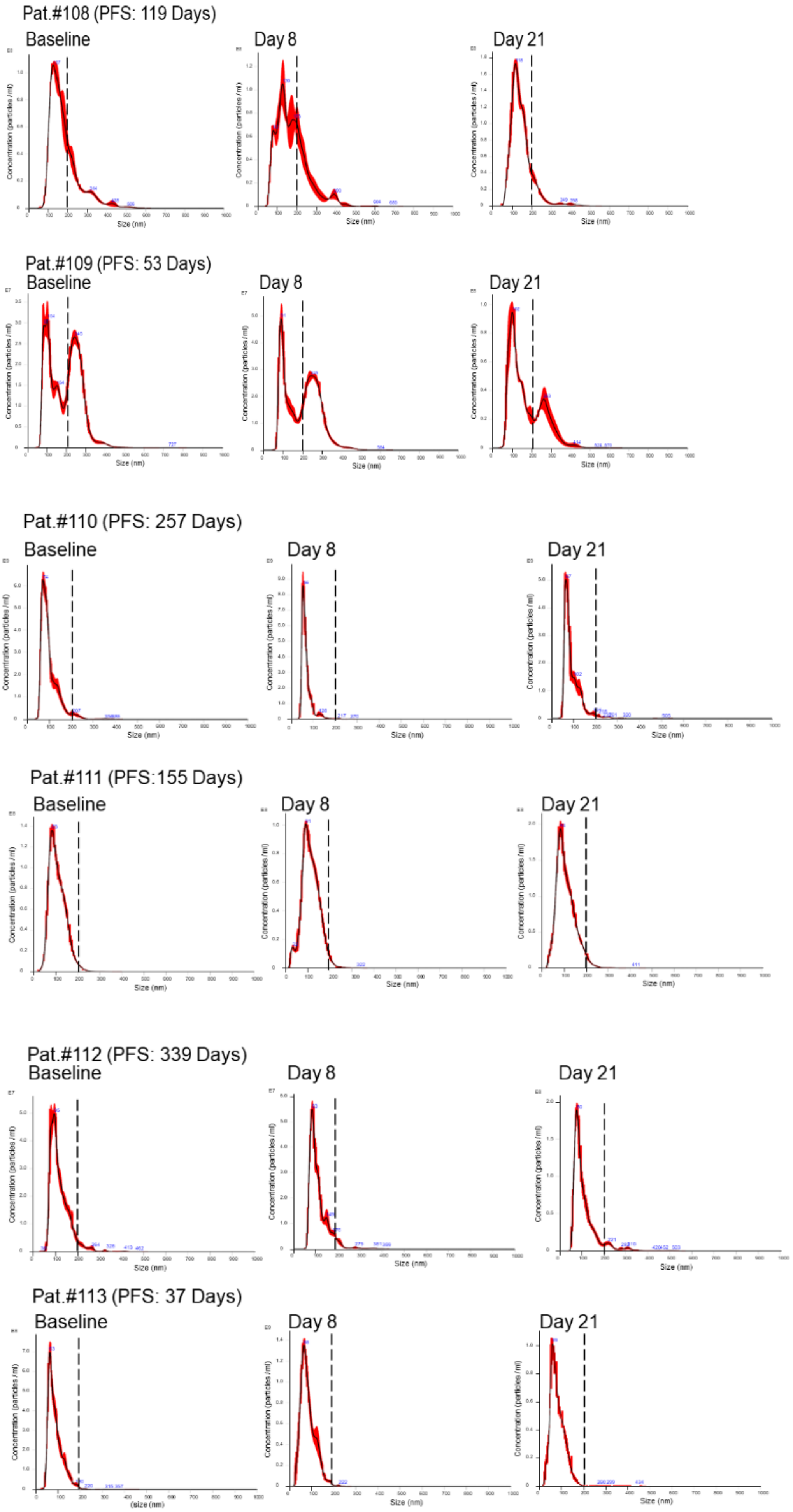
Pat.#108 (PFS: 119 Days)
Baseline
Day 8
Day 21
Pat.#109 (PFS: 53 Days)
Baseline
Day 8
Day 21
Pat.#110 (PFS: 257 Days)
Baseline
Day 8
Day 21
Pat.#111 (PFS:155 Days)
Baseline
Day 8
Day 21
Pat.#112 (PFS: 339 Days)
Baseline
Day 8
Day 21
Pat.#113 (PFS: 37 Days)
Baseline
Day 8
Day 21

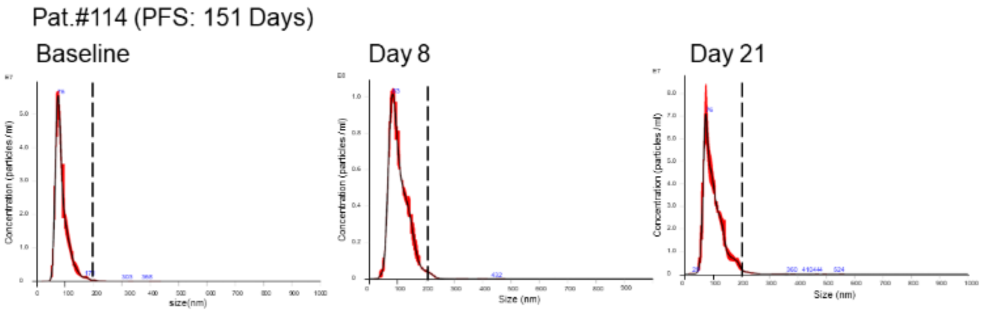

Supplementary Figure 2. Nanoparticle Tracking Analysis of Extracellular Vesicles. Extracellular vesicles (EVs) were isolated from plasma samples at baseline, day 8 and 21. EVs sizes were monitored by Nanoparticle Tracking Analysis (NTA) as described in **Figure 1**. The dotted vertical line in the graphs is set at 200 nm. Progression Free Survival (PFS) in days is stated.

Supplementary Figure 3

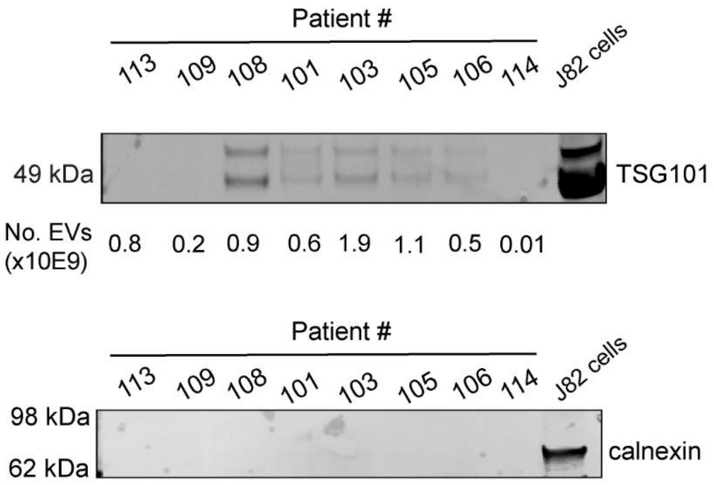

Supplementary Figure 3. Western blot profiling of Extracellular Vesicles from metastatic urothelial cancer (mUC) patient plasma at baseline. Extracellular vesicles (EVs) isolated from plasma samples at baseline was profiled by Western blotting for TSG101 and calnexin. The amount of EVs analysed with respect to TSG101 is given. Please note that normalisation for amount of EVs was not performed. The presented result just verifies that TSG101 is expressed in some but not all samples. A cell extract from the urothelial carcinoma (UC) cell line J82 was used as positive control.

Supplementary Figure 4

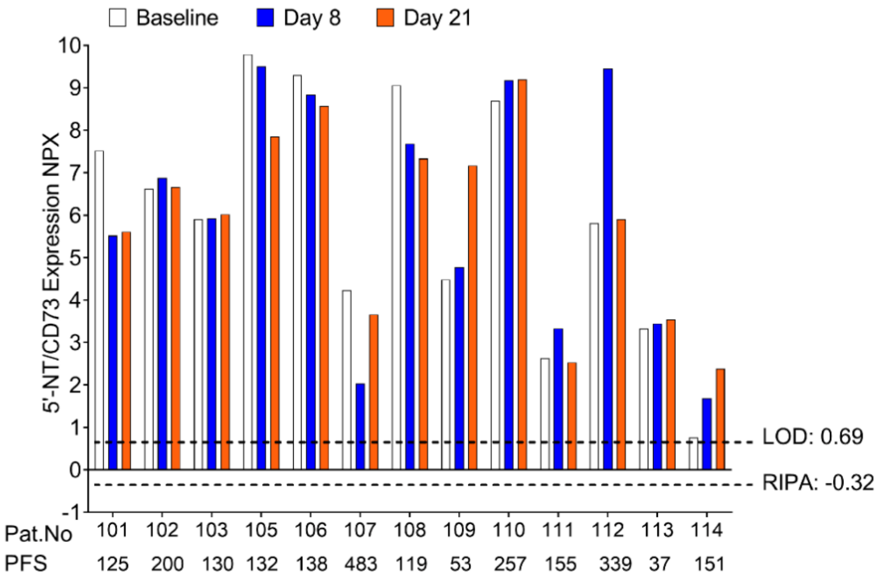

Supplementary Figure 4. Expression of CD73/5′-nucleotidase (5′-NT) in Extracellular vesicles isolated from metastatic urothelial cancer (mUC) patient plasma. Extracellular vesicles (EVs) isolated from plasma samples of metastatic urothelial cancer (mUC) patients at baseline, day 8 and 21 were subjected to Proximity Extension Assay (PEA) protein profiling with the Oncology II® assay. Expression (given as Normalized Protein eXpression (NPX) values) of CD73/5′-nucleotidase (5′-NT) in EVs from different patients is shown. No adjustment for the interpatient differences in the amount of EV analysed was undertaken. The Lower Limit of Detection (LOD) is shown with a dotted line as is the RIPA negative control. The Progression Free Survival (PFS) (in days) for the patients are specified.

Supplementary Figure 5

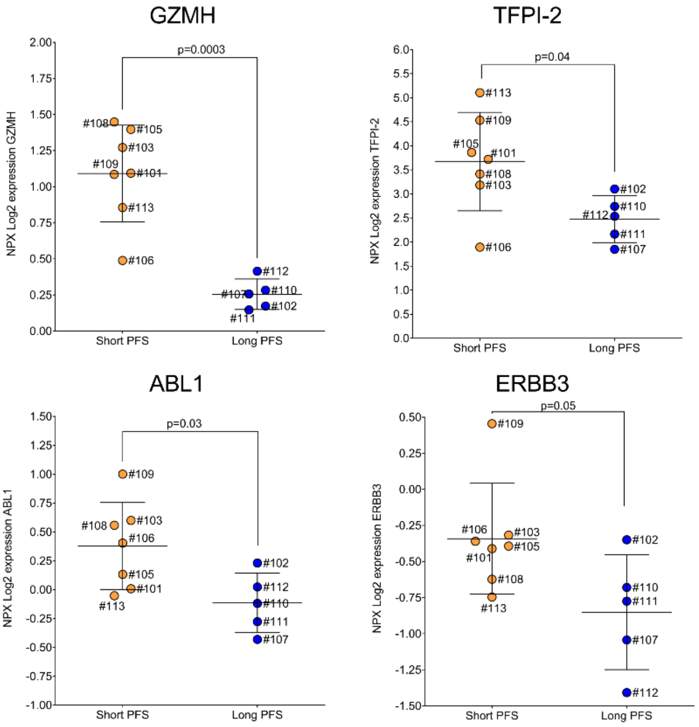

Supplementary Figure 5. **Proteins in Extracellular Vesicles at day 21 associated with Progression Free Survival (PFS)**. The Normalized Protein eXpression (NPX) expression of individual proteins analysed with Proximity Extension Assay (PEA) in extracellular vesicles (EVs) from the metastatic urothelial cancer (mUC) patients with short (≤138 days) or long (>138 days) Progression Free Survival (PFS), respectively. The proteins which only showed significant association to PFS when non-normalised PEA data was analysed are presented. No adjustment for amount of EVs was made. For Lower Limit of Detection (LOD) of the proteins see **Supplementary Table S1**. Please note that pat.#114 was excluded in the presentation of the data.

Supplementary Figure 6

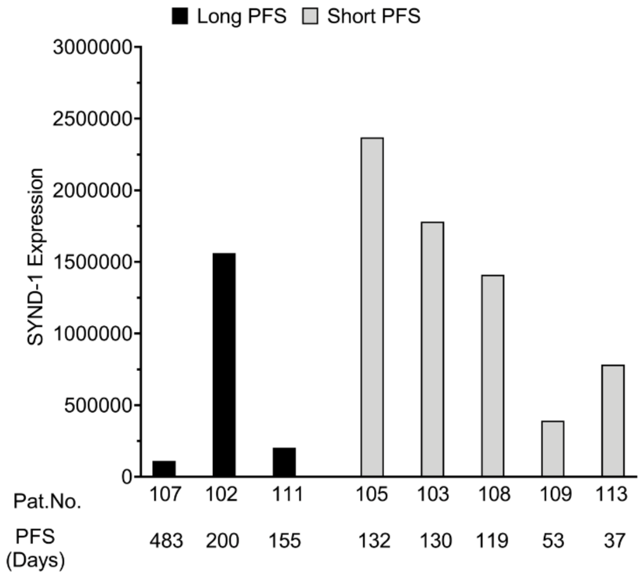

Supplementary Figure 6. **SYND-1 expression in Extracellular Vesicles at Day 21**. Densitometric quantification of SYND-1 expression in extracellular vesicles (EVs) presented in **Figure 3D** without normalisation is shown. The Progression Free Survival (PFS) in days are shown. The patients are grouped into short (≤138 days; grey bars) or long (> 138 days, black bars) PFS.
